# Stability and Transfer of *Fusarium* Mycotoxins During Oat Processing: A Review

**DOI:** 10.3390/foods15193429

**Published:** 2026-09-25

**Authors:** Irene Teixido-Orries, Francisco Molino, Carol Verheecke-Vaessen, Sheryl A. Tittlemier, Sonia Marín

**Affiliations:** 1 Applied Mycology Unit, Department of Food Technology, Engineering and Science, University of Lleida, AGROTECNIO-CERCA Centre, Av. Rovira Roure 191, 25198 Lleida, Spain; 2 Grain Research Laboratory, Canadian Grain Commission, 1404-303 Main St., Winnipeg, MB R3C 3G8, Canada; 3 Magan Centre of Applied Mycology, Cranfield University, Cranfield MK43 0AL, UK; c.verheecke@cranfield.ac.uk

**Keywords:** *Fusarium*, deoxynivalenol, T-2 toxin, HT-2 toxin, zearalenone, oat processing, mycotoxin stability, cereal safety

## Abstract

*Fusarium* mycotoxins are frequent natural contaminants of oats, posing significant challenges for food safety and processing. This review critically evaluates the behaviour of major *Fusarium* mycotoxins, including deoxynivalenol (DON), T-2 and HT-2 toxins, zearalenone (ZEN), fumonisins (FUMs), emerging toxins and selected modified forms, throughout oat processing and in derived products. Evidence consistently shows that the most effective mitigation occurs during early physical processing. Cleaning, sorting, and particularly dehulling can substantially reduce mycotoxin levels, with reductions exceeding 80–90% reported for some mycotoxins and processing conditions, mainly through the removal of outer grain layers and defective grains in which mycotoxins are preferentially concentrated, although this results in the concentration of toxins in by-products such as hulls and bran. Subsequent operations—including milling, flaking, and fractionation—primarily redistribute mycotoxins within product streams rather than eliminate them. Conventional thermal processes, such as steaming, kilning, baking, and porridge preparation, generally lead to limited reductions due to the high thermal stability of these compounds, whereas extrusion of oat flour can achieve high reductions under specific conditions. However, mycotoxins can persist and be transferred into final products, indicating that processing alone cannot ensure complete detoxification. By integrating technological, chemical, and regulatory perspectives, this review proposes a value-chain framework to support risk assessment, industrial decision-making, and future research directions for oats.

## 1. Introduction

Cereal grains are a fundamental part of the human diet worldwide, with wheat, rice and maize being the most consumed [1]. Oats (*Avena sativa* L.), although traditionally used for animal feed, currently rank seventh in global cereal production, and their role in human nutrition is expanding due to their favourable nutritional composition [2]. Oats are particularly valued for their high content of soluble dietary fibre, especially β-glucan, as well as phenolic acids, unsaturated fatty acids, minerals, and B-complex vitamins [3]. Their consumption has been associated with several health benefits, including maintenance of normal cholesterol levels, body-weight regulation, and reduced risk of type 2 diabetes and colorectal cancer, as well as potential anti-inflammatory and dermatological effects [4,5]. Consequently, oats are increasingly incorporated into a wide range of foods, including flakes, flour, biscuits, beverages, breads, oat-based pasta, and foods intended for infants and young children [6,7,8]. Oats are also commonly incorporated into gluten-free diets because they do not naturally contain gluten, although cross-contamination with gluten-containing cereals may occur during cultivation, harvesting, or processing [9]. Oat production is concentrated predominantly in temperate regions, with Central and Northern Europe representing a major producing region alongside countries such as Canada and the United States of America (USA) [10].

Despite these nutritional and technological advantages, oats, like other cereals, are susceptible to infection by *Fusarium* species, particularly during crop development [11]. *Fusarium* infection can adversely affect grain quality and yield and may result in the accumulation of mycotoxins, secondary metabolites of concern for human and animal health [12]. Available occurrence studies show that oats intended for food purposes may contain a broad range of *Fusarium* mycotoxins, with marked variability in their incidence and concentrations among studies and geographical regions (Table 1). Type A and B trichothecenes, particularly T-2 toxin, HT-2 toxin, deoxynivalenol (DON) and nivalenol (NIV), are prominent across several surveys, while zearalenone (ZEN), fumonisins (FUMs) and emerging mycotoxins such as enniatins (ENNs) and beauvericin (BEA) have also been reported [13,14]. However, direct comparisons of incidence among occurrence studies should be interpreted cautiously because the stage in the food supply chain, the range of mycotoxins investigated, and the analytical limits of quantification (LOQ) differ among surveys. Modified forms further increase the complexity of the mycotoxin profile in oats. Variations in the chemical structure and toxicity of these compounds result in different toxicological profiles and adverse health effects following dietary exposure [15]. 

Given their occurrence in oats and potential implications for consumer health, regulatory measures have been established for selected *Fusarium* mycotoxins. However, regulatory approaches differ among jurisdictions and according to the mycotoxin and oat-product category. Maximum or guidance levels relevant to oats and oat-based products in the European Union (EU), Northern Ireland, England, Scotland, Wales, Canada, and the USA are summarised in Table 2 [16,17,18,19,20,21,22].

Processing represents an important determinant of the final mycotoxin content of oat-based foods because successive operations may remove contaminated grain fractions, redistribute mycotoxins among processing streams, alter their concentrations, or promote their transformation into modified or degradation products. Although numerous studies have investigated the occurrence of *Fusarium* mycotoxins in raw oats and oat-based products, evidence concerning their behaviour throughout successive processing operations remains fragmented across different mycotoxins, processes, and experimental conditions. 

The novelty of the present review lies in integrating this dispersed evidence across the oat-processing value chain, from reception and primary processing of raw oats to secondary processing and the manufacture of final oat-based foods. Rather than considering reported changes in mycotoxin concentrations at individual processing steps in isolation, the available evidence is critically evaluated according to the fate of the mycotoxins, with particular attention to their physical removal from edible fractions, redistribution among processing streams, persistence during processing, and chemical transformation. The review further integrates major *Fusarium* mycotoxins with their relevant modified forms and emerging mycotoxins, while considering processing-related transfer into by-product streams and the potential implications for exposure. By identifying cross-process patterns and critical evidence gaps, this value-chain synthesis aims to provide an integrated understanding of *Fusarium* mycotoxin behaviour during oat processing and to support risk assessment and decision-making by oat processors and food manufacturers.

**Table 1:** Incidence, maximum value, and country of detection of mycotoxins produced by *Fusarium* in oat grains intended for food reported in different studies. DAS: diacetoxyscirpenol; DON-3G: deoxynivalenol-3-glucoside; 3-ADON: 3-acetyldeoxynivalenol; 15-ADON: 15-acetyldeoxynivalenol; FB_1_: fumonisin B_1_; FB_2_: fumonisin B_2_; ENN A: enniatin A; ENN A_1_: enniatin A_1_; ENN B: enniatin B; ENN B_1_: enniatin B_1_.

Mycotoxin	Number of Samples	Incidence (% Samples Analysed > LOQ)	LOQ (µg/kg)	Maximum Reported Concentration (µg/kg)	Country	Reference
***Fusarium*** **mycotoxins: type A trichothecenes**						
T-2 toxin	208	41	50	3405	Ireland	[13]
HT-2 toxin		51	50	1102		
T-2-glucoside		19	25	997		
DAS		ND	NA	ND		
T-2 toxin	33	79	3.1	836	United Kingdom	[23]
HT-2 toxin		85	6.3	3084		
T-2-glucoside		40	12	73		
DAS		ND	25	ND		
T-2 toxin	100	12	12	321	Spain	[14]
HT-2 toxin		36	4.6	439		
T-2 toxin	93	64	10	628	Sweden	[24]
HT-2 toxin		70	10	650		
T-2 toxin	21	ND	3.3	ND	Poland	[25]
HT-2 toxin		5	3.3	30		
T-2 toxin	31	61	4.2	1830	Finland	[26]
HT-2 toxin		74	9.0	548		
T-2 toxin	12	33	5.0	11	Czech Republic	[27]
HT-2 toxin		58	5.0	28		
T-2 toxin	325	72	20–30	4880	Switzerland	[28]
HT-2 toxin		56	30–80	3789		
DAS		ND	NA	ND		
T-2 toxin	287	7	30	108	Canada	[29]
HT-2 toxin		6	60	256		
T-2 toxin	281	95	5.0	302	Germany	[30]
HT-2 toxin		98	5.0	1000		
DAS		3	5.0	13		
***Fusarium*** **mycotoxins: type B trichothecenes**						
DON	208	2	100	330	Ireland	[13]
DON-3G		1	12	115		
NIV		2	100	338		
DON	33	45	25	2988	United Kingdom	[23]
DON-3G		24	12	881		
NIV		60	12	1253		
DON	100	22	18	736	Spain	[14]
3-ADON		3	9.0	43		
DON	93	93	13	5544	Sweden	[24]
NIV		94	13	1743		
DON	21	81	3.3	35	Poland	[25]
NIV		71	3.3	222		
3-ADON		38	3.3	10		
15-ADON		10	3.3	3		
DON	31	100	3.9	23,800	Finland	[26]
DON-3G		87	6.6	6600		
NIV		71	9.6	4940		
3-ADON		77	11	2720		
DON	12	ND	50	ND	Czech Republic	[27]
NIV		17	80	304		
DON	325	51	10–60	1328	Switzerland	[28]
NIV		68	20–210	1653		
DON	287	63	30	4130	Canada	[29]
DON-3G		28	50	858		
NIV		14	60	723		
3-ADON		2	50	251		
15-ADON		ND	50	ND		
DON	281	91	10	1414	Germany	[30]
DON-3G		63	10	195		
NIV		91	10	596		
3-ADON		54	10	193		
15-ADON		17	10	65		
***Fusarium*** **mycotoxins: FUMs**						
FB_1_	100	6	60	217	Spain	[14]
FB_2_		3	41	74		
FB_1_ + FB_2_	12	8	30	19	Czech Republic	[27]
FB_1_	287	0.3	50	79	Canada	[29]
FB_2_		ND	50	ND		
***Fusarium*** **mycotoxins: ZEN**						
ZEN	208	1	20	30	Ireland	[13]
	100	37	28	153	Spain	[14]
	93	22	5.0	2297	Sweden	[24]
	31	42	5.7	675	Finland	[26]
	287	5	20	169	Canada	[29]
	12	ND	20	ND	Czech Republic	[27]
	281	16	5.0	47	Germany	[30]
**Emergent mycotoxins of *Fusarium***						
BEA	208	3	25	150	Ireland	[13]
ENN A_1_		9	50	347		
ENN B		24	100	1517		
ENN B_1_		13	100	1021		
BEA	93	99	1.7	327	Sweden	[24]
ENNs		100	6.8	862		
BEA	12	33	5.0	35	Czech Republic	[27]
ENN A_1_		17	5.0	5		
ENN B		33	5.0	17		
ENN B_1_		17	5.0	15		
BEA	287	94	2.0	271	Canada	[29]
ENN A		11	2.0	18		
ENN A_1_		12	5.0	37		
ENN B		45	16	1332		
ENN B_1_		13	16	301		

**Table 2:** Regulatory and guidance levels for the main *Fusarium* mycotoxins relevant to oats and oat-based products in selected jurisdictions [16,17,18,19,20,21,22]. Values are expressed in µg/kg. — indicates that no maximum or guidance level has been established for the specified product/category in the jurisdiction considered.

Mycotoxin	Product/Category	European Union (EU) and Northern Ireland (µg/kg)	England, Scotland and Wales (µg/kg)	Canada/United States of America (USA) (µg/kg)
**T-2 + HT-2 toxins**	Unprocessed oats with husk	1250	—	—
	Oats placed on the market for the final consumer	100	—	—
	Oat milling products (including oat bran), oat flakes and bakery wares containing ≥90% oat milling products	100	—	—
	Other bakery wares, pasta and cereal snacks	20	—	—
	Breakfast cereals meeting specified high-cereal criteria, <40% oat products/whole oats	50	—	—
	Breakfast cereals meeting specified high-cereal criteria, ≥40% oat products/whole oats	75	—	—
	Baby food and processed cereal-based foods for infants/young children	10	—	—
**DON**	Unprocessed oats with inedible husk	1750	1750	—
	Cereals placed on the market for the final consumer	750	750	—
	Milling products	600	750	—
	Bakery wares, cereal snacks and breakfast cereals	400	500	—
	Pasta	600	750	—
	Baby food and processed cereal-based foods for infants/young children	150	200	—
**ZEN**	Unprocessed oats with husk	100	100	—
	Cereals, bran and germ for final consumer; cereal flour/semolina	75	75	—
	Bread, pastries, biscuits, cereal snacks and breakfast cereals	50	50	—
	Baby food and processed cereal-based foods for infants/young children	20	20	—
**FUMs**	Oats and oat-based products	—	—	—
**ENNs/BEA**	Oats/oat-based foods	—	—	—

### 1.1. Fusarium Mycotoxins

*Fusarium* is one of the predominant fungal genera associated with oats [28] and is the causal agent of Fusarium head blight (FHB). Although oats are generally less susceptible to FHB than other cereals such as wheat and barley, partly due to the presence of long pedicels between spikelets that restrict fungal spread, high levels of *Fusarium* mycotoxins may still occur [31]. The occurrence and resulting profiles of these mycotoxins are strongly influenced by environmental conditions and the composition of *Fusarium* species populations [32,33]. 

As reflected in Table 1, the relative occurrence of individual *Fusarium* mycotoxins varies considerably among oat-producing regions. Studies from Sweden, Finland, Poland, Norway, Canada and Spain have frequently reported DON and related compounds, together with NIV and ZEN, whereas type A trichothecenes and their modified forms have been particularly prominent in studies from the United Kingdom (UK), Ireland, the Czech Republic and Switzerland [13,14,23,24,25,26,27,28,29,34]. Emerging *Fusarium* mycotoxins may also occur frequently and co-occur with the better-characterised toxin groups [13,24,27,35]. These geographical patterns should not, however, be interpreted as fixed regional profiles, as they reflect differences in agroclimatic conditions, harvesting years, cropping systems and, importantly, the *Fusarium* species populations prevailing in each production environment. Changes in climatic conditions may further alter species distribution and, consequently, the mycotoxin profiles observed in oats [33].

#### 1.1.1. Trichothecenes

Trichothecenes are sesquiterpenoid mycotoxins characterised by a 12,13-epoxytrichothec-9-ene core structure and are classified into four types (A, B, C and D) [36]. Among them, types A and B are considered the most relevant from a food safety perspective in cereals due to their frequent occurrence and pronounced toxicological effects and are therefore described in the following sections.

##### Type A Trichothecenes 

Type A trichothecenes are of particular relevance in oats, where T-2 (Figure 1(**1**)) and HT-2 (Figure 1(**2**)) toxins are among the most frequently reported *Fusarium* mycotoxins [37]. They are mainly associated with species such as *F. sporotrichioides* and *F. langsethiae*, although other *Fusarium* species may also contribute to their occurrence [33]. Other type A trichothecenes detected in oats include neosolaniol (NEO) (Figure 1(**3**)) and diacetoxyscirpenol (DAS) (Figure 1(**4**)), together with metabolites and modified forms such as T-2 triol, T-2 tetraol, T-2-glucoside and HT-2-glucoside [38]. These metabolites and modified forms are particularly relevant to processing because transformations among different toxin forms may influence their apparent reduction, persistence, and overall fate in processed oat products.

Type A trichothecenes are potent inhibitors of protein synthesis and are associated with gastrointestinal, immunological, and other adverse effects [38,39,40]. The European Food Safety Authority (EFSA) has established a group tolerable daily intake (TDI) of 0.02 µg/kg body weight/day for the sum of T-2 and HT-2 toxins and their modified forms [38]. Binding maximum levels for T-2 and HT-2 toxins are established in the EU and in Northern Ireland, with values varying according to the oat-product category (Table 2) [16]. In contrast, no maximum levels have been established in Canada, USA, Wales, Scotland, and England [19,20,21,22,41]. 

##### Type B Trichothecenes 

Type B trichothecenes are also highly relevant in oats, particularly DON (Figure 2(**5**)) and, to a lesser extent, NIV [24,26,33]. DON is primarily associated with *F. graminearum* and *F. culmorum*, and contaminated oats may also contain DON-related forms such as deoxynivalenol-3-glucoside (DON-3G) (Figure 2(**6**)), 3-acetyl-deoxynivalenol (3-ADON) (Figure 2(**7**)), and 15-acetyl-deoxynivalenol (15-ADON) (Figure 2(**8**)) [42,43,44,45]. The acetylated derivatives 3-ADON and 15-ADON originate from fungal biosynthesis, whereas DON-3G is formed through plant detoxification by glucosylation [45]. Although these forms generally occur at lower concentrations than DON, they are toxicologically relevant because they may contribute to overall DON exposure, including through conversion to DON during digestion. They are also particularly relevant to processing, as changes in their concentrations or interconversion with DON may influence the apparent fate of the parent toxin.

Processing may additionally generate or alter DON-related transformation products, including isodeoxynivalenol (isoDON), nordeoxynivalenol (norDON) and deepoxy-deoxynivalenol (DOM-1), which have been reported in oats [46]. Unlike DON and its acetylated derivatives, these compounds are not considered primary *Fusarium* metabolites and may arise through degradation or structural rearrangement, particularly under thermal or alkaline processing conditions [47,48]. Their occurrence is therefore relevant when interpreting apparent reductions in DON.

Type B trichothecenes primarily inhibit protein synthesis and are associated with gastrointestinal and immunological effects following dietary exposure [49,50]. EFSA has established a group TDI of 1 µg/kg body weight/day for the sum of DON, 3-ADON, 15-ADON and DON-3G, reflecting their combined contribution to DON exposure [51]. Maximum levels for DON in oats and oat-based products are established in the EU and in Northern Ireland, whereas regulatory approaches differ in Wales, Scotland, England, Canada, and the USA (Table 2) [19,20,21,22]. Current legal EU maximum levels apply to DON rather than to its modified forms, despite their consideration within the EFSA group TDI.

#### 1.1.2. Zearalenone (ZEN)

ZEN (Figure 3(**9**)) is an oestrogenic *Fusarium* mycotoxin occurring in oats, mainly associated with *F. graminearum* and *F. culmorum* [52,53]. In addition to the parent toxin, reduced metabolites such as α-zearalenol (α-ZEL) and β-zearalenol (β-ZEL) may occur, with α-ZEL exhibiting greater oestrogenic potency than ZEN [54]. ZEN and its modified or transformation products are relevant to processing because their relative concentrations may change during thermal and other treatments. ZEN acts primarily through interaction with oestrogen receptors and has been associated with reproductive and endocrine effects [55,56]. Regulatory limits applicable to ZEN in oats and oat-based products are summarised in Table 2 [18].

#### 1.1.3. Fumonisins (FUMs)

FUMs, particularly FB_1_ (Figure 4(**10**)) and FB_2_, are primarily associated with maize but have occasionally been detected in oats [18,29]. They are mainly produced by *F. verticillioides* and *F. proliferatum*, with FB_1_ generally representing the predominant and toxicologically most relevant analogue [57,58,59]. FUMs disrupt sphingolipid metabolism and have been associated with several adverse health effects, including hepatotoxicity and carcinogenicity [60,61]. Their occurrence and behaviour during oat processing remain considerably less characterised than those of DON, T-2 and HT-2 toxins, and ZEN. Although regulatory limits for FUMs have been established for certain commodities, particularly maize-based products, no oat-specific maximum levels have been established in the jurisdictions considered in this review (Table 2).

#### 1.1.4. Emerging *Fusarium* Mycotoxins

Emerging mycotoxins are generally defined as fungal secondary metabolites that are not yet regulated and are not routinely included in mycotoxin monitoring programmes [62]. Several emerging *Fusarium* mycotoxins have been reported in oats, including BEA (Figure 5(**11**)), ENNs, CUL, fusaric acid and fusarenon-X [13,27,29,35,63,64]. Of these, ENNs (ENN A (Figure 5(**12**)), ENN A_1_ (Figure 5(**13**)), ENN B (Figure 5(**14**)), and ENN B_1_ (Figure 5(**15**))) and BEA are frequently detected and may co-occur with regulated *Fusarium* mycotoxins [65]. Although toxicological information remains limited, available studies indicate potential intestinal, immunological, and reproductive effects [66]. Importantly, information on their stability, redistribution, and transformation during oat processing remains particularly scarce, limiting the assessment of their fate and potential contribution to exposure in processed oat products. No maximum levels have been established for these compounds in the jurisdictions considered in this review (Table 2).

## 2. Oat and Oat-Based Products Processing

As outlined in the previous Section, oats are frequently contaminated with *Fusarium* mycotoxins, which may persist along the cereal supply chain if not effectively mitigated at pre-harvest, harvest, or post-harvest stages [67]. Consequently, these contaminants can be transferred into oat-derived food products, contributing to consumer exposure [7,68,69,70]. A clear understanding of the principles and objectives of oat processing operations is therefore required to accurately assess how different processing steps influence the stability, redistribution, and potential reduction in *Fusarium* mycotoxins.

Oat processing is a well-structured, multistep industrial operation designed to convert raw harvested grain into a wide range of stable, nutritious, and functional food products, as shown in Figure 6. This transformation is achieved through a series of physical, thermal, and enzymatic interventions, each of which plays a strategic role in modifying the grain’s properties while ensuring food safety and technological viability. The process includes not only cleaning, dehulling, and heat treatment, but also specialised operations such as enzymatic hydrolysis, flaking, milling, and fractionation to produce ingredients suited for oat-based products such as infant cereals, beverages, and baked goods.

As described by Menon et al. [8], processing begins with oat crop harvest, which is typically carried out using combine harvesters once the grain reaches physiological maturity. Timely harvest is critical to prevent issues like pre-harvest sprouting or fungal infection. After harvesting, oats are transported in bulk to processing facilities, where care is taken to prevent mechanical damage or moisture gain during transport.

Upon arrival, the lots are subjected to reception, inspection, and weighing. This stage allows verification of quality parameters such as moisture content and grain uniformity, as well as the presence of foreign material, damaged grains, or other visible defects. Rapid mycotoxin screening may also be performed using field-deployable tools such as lateral flow devices (LFDs) or immunoassays. This represents an important control point, as early identification of lots with elevated mycotoxin concentrations can prevent unnecessary downstream handling and processing of grain that may not meet regulatory or internal acceptance criteria. Oat lots intended for processing in the EU must comply with the maximum levels established by the European Commission for unprocessed cereals with respect to DON, the sum of T-2 and HT-2 toxins, and ZEN [16,17,18]. Moreover, EU legislation also specifies sampling and analytical requirements for the official control of mycotoxins in food [71].

Following acceptance, oats can enter storage, typically in silos with aeration systems designed to maintain stable temperature and relative humidity. Effective storage conditions mitigate the risks of spoilage, condensation, insect infestation, and microbial growth, allowing safe preservation of the raw material until needed [72].

The first major transformation step is cleaning and sorting, during which the oat stream is freed of stones, dust, straw, weed seeds, and broken or discoloured grains [73]. Sieving or screening based on particle and grain size, together with aspiration, is used to remove foreign material and undesirable grain fractions, while optical sorters may be employed for the targeted removal of defective grains. At this point, oats are still whole grains with the hull intact.

Dehulling is then carried out to remove the inedible fibrous husk from the inner kernel, which is known as groat [74]. The groat is the true edible core of the oat grain and comprises the starchy endosperm, the germ, and the bran layers. Dehulling is conducted using impact- or friction-based machines, and scouring may follow to polish the groats and remove residual hull fragments or microbial surface contaminants (this fraction could also be used as bran or feed). Effective hull removal is important for product quality and downstream processing efficiency. Hulls can also be processed to obtain oat fibre, mainly intended to produce animal feed [75]. 

Once isolated, groats undergo kilning, a dry-heat treatment that further stabilises the groats and enhances flavour [76]. This is immediately followed by steaming, which softens the groats and inactivates endogenous enzymes such as lipase that could cause rancidity [77]. This process includes the injection of steam that increases temperature and moisture. After these processes, the stabilised groats can proceed to various finishing lines depending on the final product.

The flaking process is one of the most important steps in oat milling, as it transforms oat groats into rolled oats, which are widely used in food products [7,78]. There are four main types of oat flakes, which differ in size and thickness: whole rolled oats, quick oats, instant oats, and steel-cut oats. After steaming, the steamed groats are then passed through a pair of smooth rollers that press them into flakes. The space between the rollers can be adjusted to control flake thickness, which determines the final product type. After rolling, the flakes are cooled and dried in a fluidised bed to avoid sticking or clumping. Finally, a sifting step removes broken flakes or oversized pieces to ensure a uniform product.

Oat bran, another important product, is obtained by sieving and separating coarse fractions during flour milling [8]. Unlike wheat bran, oat bran is not a defined anatomical layer but rather a fraction enriched in β-glucan and dietary fibre. It is used in functional foods and health-targeted formulations.

Processed groats or flakes can then be milled to produce oat flour, which may serve as an ingredient in bakery products, snacks, extruded foods, or several formulations [78]. Oat flour is usually obtained by grinding kilned groats or rolled oats via hammer mills. Whole oat flour is defined as being derived entirely from groats, retaining all fractions of the grain.

In some operations, oat flour is subjected to roasting and enzymatic treatment, particularly in the production of infant cereals [79]. These involve thermal pre-treatment followed by enzymatic hydrolysis using α-amylases, reducing viscosity and increasing digestibility. The final product is a dried powder with rapid solubility and low microbial load, suitable for reconstitution.

As explained by Schwake-Anduschus et al. [80], oat porridge (called oatmeal in some countries) is traditionally prepared from rolled or steel-cut oats by boiling them in water or milk. Flake thickness and the degree of starch gelatinisation, i.e., the extent to which starch has already undergone structural changes during previous thermal processing, influence water absorption and therefore the cooking time required to obtain the desired texture. 

Other products, such as oat-based beverages (OBBs), are produced by enzymatically hydrolysing oat flour or flakes in water (liquefaction process), followed by filtration or decantation, homogenisation, thermal treatment (e.g., sterilisation or pasteurisation) and cooling. The resulting liquid may be fortified, flavoured, or stabilised and is typically packaged in aseptic containers [81]. This product is increasingly popular as a dairy alternative and base for oat yoghurts, cream, or ice cream, among others [4]. 

Oat-based bakery products, including biscuits, muffins, and breads, integrate oat flour, flakes, or bran into their formulations [82]. Oat ingredients contribute fibre, moisture retention, and health benefits, though adjustments in structure and leavening are often needed due to the lack of gluten [83]. The usual process includes mixing ingredients, moulding, baking, cooling, and packaging. Different baked products include granola and crunchy muesli, whose production involves mixing oat flakes with sugars, fats, and other inclusions, followed by toasting or baking at high temperatures. 

Additional items like oat pasta and noodles are emerging products in the functional food segment. These are prepared by mixing oat flour (or blends including oat bran) with water and often hydrocolloids such as guar or xanthan gum to compensate for the lack of gluten. The dough is extruded or sheeted, cut into strands, and dried [84]. 

Against this background, the following Sections critically review the available evidence on the stability and transfer of *Fusarium* mycotoxins during the processing of oats and oat-based products, highlighting current limitations and knowledge gaps.

## 3. *Fusarium* Mycotoxins Behaviour During Oat Processing

Although numerous studies have reported the occurrence of these toxins in raw grains and final products, a systematic and critical understanding of how successive processing steps influence their stability, redistribution, transformation, and transfer throughout the oat value chain remains limited. Therefore, this review provides a comprehensive evaluation of the behaviour of major *Fusarium* mycotoxins—DON, T-2 and HT-2 toxins, ZEN, relevant modified forms, and emerging mycotoxins—during both primary and secondary oat processing. Primary processing comprises operations applied to raw oat grain to obtain edible oat fractions and ingredients, including cleaning and sorting, dehulling and scouring, hydrothermal treatment, and milling and flaking. Secondary processing involves the subsequent transformation of these oat fractions and ingredients into oat-based foods through processes such as extrusion, baking, porridge preparation, and oat-based beverage manufacture.

### 3.1. Oat Reception, Cleaning and Sorting

The initial stages of oat processing, particularly reception, cleaning and sorting, are important control points for managing *Fusarium* mycotoxins. In this context, cleaning refers primarily to the removal of foreign and undesirable material from the incoming oat stream, whereas sorting involves the separation of oat kernels according to physical characteristics, allowing damaged, discoloured, small or lightweight kernels to be separated from sound kernels. Since *Fusarium* mycotoxins are often unevenly distributed within oat consignments and may be concentrated in these lower-quality fractions, their removal can reduce the mycotoxin burden of the material entering subsequent processing stages.

Reception controls aim to assess whether incoming oat consignments comply with regulatory and quality requirements. However, such assessments cannot fully account for the pronounced heterogeneity of *Fusarium* mycotoxin contamination within a consignment. Cleaning and sorting therefore represent the first active processing interventions capable of reducing mycotoxin concentrations through physical separation of foreign material and more highly contaminated kernel fractions [73]. In addition to conventional separation based on size, density, and other physical characteristics, spectroscopic approaches have recently been investigated as tools to support decision-making at this stage. Near-infrared hyperspectral imaging (NIR-HSI), for example, does not directly measure mycotoxins but captures spectral characteristics of the grain that can be associated with differences in mycotoxin concentration. Teixido-Orries et al. [85,86] demonstrated that NIR-HSI models could classify oat samples at delivery according to whether they exceeded predefined mycotoxin thresholds, achieving maximum classification accuracies of 86–94% for the 1000 µg/kg threshold for the sum of T-2 and HT-2 toxins and the 1750 µg/kg threshold for DON. 

Teixido-Orries et al. [87,88] further investigated the marked within-sample heterogeneity of *Fusarium* mycotoxin contamination at the individual-grain level. Their findings showed that a relatively small proportion of highly contaminated grains can contribute disproportionately to the mycotoxin concentration of an oat consignment. Combining individual-grain assessment with NIR-HSI-based sorting could therefore provide a means of identifying and selectively removing grains associated with a higher mycotoxin concentration. Such an approach could reduce mycotoxin concentrations in the retained oat fraction while avoiding unnecessary rejection of otherwise usable material, thereby improving food safety while reducing raw-material losses.

A particularly detailed evaluation of size-based sorting was reported by Brodal et al. [89], who investigated the effect of removing small oat grains on mycotoxin contamination in naturally contaminated oat lots from two harvest years. Using a 2.2 mm sieve, the authors separated grain into large and small grain fractions and demonstrated that the removal of the small grain fraction, representing on average 15–21% of the total grain mass, resulted in substantial reductions in several *Fusarium* mycotoxins. Mean concentrations of T-2 and HT-2 toxins were reduced by 32–56%, while DON concentrations decreased by up to 24%. In addition, marked reductions were observed for ENNs, particularly ENN B, which decreased by 44%, and a general trend towards lower levels of ADON, NIV, ENNs, and BEA was observed following the removal of small grain. Importantly, the small grain fraction itself consistently contained much higher toxin concentrations than the unsorted grain, with T-2 toxin and HT-2 toxin levels up to 272% higher, indicating that *Fusarium* infection and mycotoxin accumulation are strongly associated with reduced grain size and weight. In addition, Meyer et al. [90] conducted a study in which they separated large grains from smaller ones prior to dehulling. They also observed that smaller grains tended to contain higher concentrations of T-2 and HT-2 toxins. Specifically, they found that large grains had an average concentration of 44 μg/kg, while the smaller grains showed a significantly higher concentration of 105 μg/kg. For DON, no statistically significant differences were observed between large and thin grains, although higher concentrations tended to occur in thin grains.

A detailed study by Pettersson et al. [91] demonstrated that the combination of cleaning, optical sorting, and dehulling can achieve substantial reductions in T-2 toxin and HT-2 toxin levels in oats, with 80–95% reduction observed in highly contaminated batches. In contrast, samples with lower initial contamination exhibited more modest reductions, indicating that the efficacy of these processes is proportional to the starting toxin load. Sorting and sieving to remove debris and small grains (<2 mm) markedly reduced the levels of T-2 and HT-2 toxins in the kernels for further processing compared to the harvested oat. The application of colour sorting further enhanced safety by removing discoloured grains, which were found to contain up to 10 times more toxins than visually sound ones.

Moreover, Hietaniemi et al. [73] investigated the effect of oat sorting on trichothecene contamination in oats, focusing on DON, 3-ADON, T-2 toxin and HT-2 toxin. Pilot-scale trials demonstrated that sorting alone substantially reduced mycotoxin levels, particularly through the removal of light grains and impurities using high air-volume separation systems. DON and 3-ADON concentrations were reduced to approximately one-half or one-third of their initial levels, while T-2 toxin and HT-2 toxin concentrations decreased to about two-thirds of the original content. Notably, these reductions were achieved with a minimal loss of grain mass (approximately 2%, corresponding to 10% by volume), highlighting the high efficiency of this intervention. The authors further showed that sorting was especially effective for toxins associated with early-season *Fusarium* infection, indicating that physical separation preferentially removes kernels most severely affected during early stages of crop development.

Regarding mechanical cleaning on naturally contaminated oat samples, Schwake-Anduschus et al. [80] evaluated its effect using a laboratory-scale grain cleaner and found that the process had a highly variable impact on T-2 toxin and HT-2 toxin levels. While some samples showed moderate reductions of 10–15%, particularly in batches with higher initial contamination, other samples exhibited no reduction at all, and in some cases, toxin levels remained unchanged or even increased slightly after cleaning. Conversely, a few individual cases demonstrated substantial efficacy, with one sample showing a complete reduction, and another achieving a 69% decrease in total toxin content. This lack of consistency may be attributed to the limited physical removal capacity of the equipment and the heterogeneous distribution of toxins within grain fractions. 

In a full-scale industrial oat flake processing line, Scudamore et al. [92] investigated the fate of T-2 toxin, HT-2 toxin, DON, and NIV. In this study, optical colour sorting was applied at the groat level, following dehulling and kilning, and therefore represents a downstream classification step rather than a primary sorting operation at oat reception. Colour sorting separated a fraction of visually discoloured groats, classified as rejects and representing approximately 10–15% of the groat stream. These discoloured groats consistently exhibited higher concentrations of each mycotoxin than the accepted groats, confirming a clear association between visual defects and contamination. However, mass balance analysis showed that mycotoxins were distributed across multiple processing fractions, including oat flakes, pellets, and rejects, and that colour sorting primarily resulted in a redistribution of contamination rather than a uniform reduction in the final product. Accordingly, colour sorting should be regarded as a targeted segregation step that concentrates highly contaminated groats into a removable fraction, rather than as a stand-alone mitigation strategy.

Finally, Dropa et al. [7] conducted a detailed industrial-scale study to track the evolution of mycotoxin levels throughout the oat flake production process. Their analysis demonstrated that cleaning and dehulling constituted the most impactful mitigation step, achieving reductions of approximately 70–80% for ENN B, 75–87% for ENN B_1_, 48–100% for HT-2 toxin, 58–80% for T-2 toxin, and 35–65% for BEA. These values underscore the crucial role of early-stage mechanical interventions in mitigating contamination. The authors emphasised that these steps primarily act through physical removal of outer grain fractions and damaged grains, where mycotoxins tend to accumulate.

Taken together, these results demonstrate that cleaning and sorting can reduce *Fusarium* mycotoxins depending on the methods used and the initial level of contamination. The most effective reductions are obtained when multiple operations are combined, including small grain removal, colour sorting, and cleaning. 

### 3.2. Storage

Although *Fusarium* species are primarily associated with field infection, post-harvest storage conditions may influence both the persistence of *Fusarium* mycotoxins already present in oats and the potential for further toxin production when conditions permit fungal activity. Their behaviour during storage is strongly influenced by environmental variables such as temperature, water activity (a_w_), storage duration, and substrate composition [93].

T-2 toxin and HT-2 toxin are primarily produced by *F. langsethiae* and *F. sporotrichioides.* In oat grains, Mylona and Magan [72] demonstrated that the highest levels of T-2 toxin and HT-2 toxin were produced when grains inoculated with *F. langsethiae* were stored at 25 °C and 0.97 a_w_, with T-2 toxin exceeding 4 mg/kg and HT-2 toxin reaching 0.8 mg/kg after just 10 days. These conditions also resulted in significant dry matter losses, confirming active fungal metabolism. The authors proposed that monitoring CO_2_ production during storage could be a useful early indicator of spoilage and mycotoxin build-up. Medina and Magan [94] further confirmed that *F. langsethiae* strains from across northern Europe produced T-2 toxin and HT-2 toxin most abundantly between 20 and 30 °C and 0.98 and 0.995 a_w_, with toxin biosynthesis sharply dropping below 0.93 a_w_. Kokkonen et al. [95] observed that *F. sporotrichioides* produced maximum levels of type A trichothecenes at 15 °C, while *F. langsethiae* peaked at 25 °C, both under high water activity (0.98–0.995 a_w_). Importantly, these experiments were conducted under high-moisture conditions deliberately favourable to fungal growth and toxin production and should not be considered representative of properly managed oat storage. Canadian safe-storage guidance for oats illustrates that allowable grain moisture decreases as storage temperature increases, from approximately 17% at 0 °C to 8% at 21 °C, highlighting the importance of maintaining an appropriate temperature–moisture combination to prevent spoilage [96]. Rather, they demonstrate the potential for substantial post-harvest T-2 and HT-2 toxin production when moisture control fails.

Evidence on DON behaviour during commercial oat storage is limited; however, Tittlemier et al. [29] provided relevant observational data from Canadian oats collected at harvest and from subsequent deliveries to processors and train shipments. DON was detected more frequently in delivery and shipment samples (81%) than in harvest samples (63%), whereas highly contaminated samples (>1000 µg/kg) were less frequent in delivery and shipment samples (0.4%) than at harvest (6.3%). The authors suggested that this difference could partly result from compositing oats from different sources during commercial handling, which may increase the frequency of DON detection while diluting highly contaminated grain. Importantly, when DON concentrations in delivery and shipment samples were examined over time, no consistent increasing or decreasing trend was observed. Therefore, although these observational data cannot directly quantify changes occurring during storage, they provide no indication of systematic DON accumulation during commercial oat storage and handling.

Controlled studies in other cereals nevertheless demonstrate that DON, ZEN, and NIV can be produced post-harvest when environmental conditions favour fungal activity. In wheat, *F. graminearum* and *F. culmorum* have been shown to favour warm (25–30 °C) and humid conditions [97]. Homdork et al. [98] demonstrated that during long-term storage of wheat seeds (up to 36 weeks), ZEN accumulated under warm and humid conditions (25 °C, 90% relative humidity), while DON increased only in samples with low to moderate initial infection, and NIV appeared during highly humid storage despite being absent at harvest. In contrast, dry storage conditions restricted fungal activity and preserved grain quality. The importance of moisture control is also supported by studies in maize, where DON and ZEN did not increase during storage for up to six months at 12–20 °C when grains were adequately dried (≤7% moisture), and DON concentrations often decreased over time [99]. As with the experimental studies on T-2 and HT-2 toxins, these findings indicate that post-harvest *Fusarium* mycotoxin production is strongly dependent on moisture availability and that results obtained under conditions favourable to fungal growth should not be directly extrapolated to properly managed oat storage. In particular, evidence on the behaviour of DON, ZEN and NIV under representative commercial oat storage conditions remains limited, representing an important knowledge gap.

In a more applied context, Janavičienė et al. [100] investigated the behaviour of type A trichothecenes in oat flour during short- and medium-term storage under controlled temperature and humidity conditions. The authors reported pronounced reductions in T-2 toxin and HT-2 toxin concentrations after six weeks of storage, ranging from 38–93% and 12–95%, respectively, depending on storage temperature. A statistically significant decrease in T-2 toxin levels was already observed after three weeks at 20 °C; however, prolonged storage at this temperature did not result in further reductions and showed a tendency towards increasing T-2 toxin concentrations over time. In contrast, storage under cooler conditions (8 °C, 80% relative humidity) led to sustained decreases in both T-2 toxin and HT-2 toxin levels. Importantly, the study addressed initial sample heterogeneity by collecting grain from four locations within the storage area and homogenising the samples before analysis. The storage experiment was subsequently conducted using six naturally contaminated oat samples with four replicates, although the preparation and allocation of subsamples among storage conditions were not described in sufficient detail to fully assess the contribution of residual sampling variability to the observed changes in toxin concentrations.

Moreover, Pokrzywa et al. [101] evaluated HT-2 toxin contamination during storage of oat grains, oat bran, and oat-containing cereal products intended for children at 6 and 28 °C for up to 28 days. During storage, the proportion of samples positive for HT-2 toxin increased from 50% to 100% in oat grains and from 25% to 100% in oat bran at both temperatures, although changes in mean concentrations were comparatively small and did not show a consistent temperature-dependent pattern. In cereal products intended for children, HT-2 toxin was not detected before storage but was detected in all samples after 14 and 28 days, at concentrations of approximately 9–13 µg/kg. Interpretation of these findings requires consideration of the experimental design. Only eight samples of each product category were examined, and samples selected for the storage experiment had previously shown mould growth. In addition, their moisture content was adjusted to 19% by adding water before storage, thereby providing conditions favourable to fungal activity. Thus, although the study demonstrates that HT-2 toxin occurrence can change during storage under the conditions investigated, these conditions may not fully represent typical storage of oat products and limit direct extrapolation to commercial or household storage.

Building on these findings, Pokrzywa et al. [102] investigated whether supplementation with antioxidant-rich ingredients could limit HT-2 toxin formation during storage. As in the previous study, only samples considered contaminated during the initial screening were selected for the storage experiment. The experiment included one oat-grain sample, two oat-bran samples, and four cereal products intended for children from different brands. Samples were milled and supplemented with freeze-dried blueberry or cranberry, or cinnamon powder at concentrations ranging from 3% to 30%. Their moisture content was subsequently adjusted to 19%, and samples were stored at 28 °C for 14 days. The effect of supplementation on HT-2 toxin concentrations was strongly dependent on the ingredient, dose, and matrix. Blueberry and cranberry generally produced modest and variable effects, with reductions occurring mainly at the highest supplementation levels, whereas cinnamon showed a clear dose-dependent effect. At 30% cinnamon supplementation, HT-2 toxin concentrations after storage were approximately 76% lower in oat grain and 83–87% lower in oat bran than in the corresponding unsupplemented controls; concentrations also decreased markedly in all four cereal products intended for children, falling below the LOQ in two of them. These findings nevertheless require cautious interpretation. Although milling before treatment may have reduced within-sample heterogeneity, the very limited number of independent samples restricts the assessment of between-sample variability and the generalisability of the observed effects. Furthermore, the selection of previously contaminated material, together with the adjustment of moisture content to 19% to provide conditions deliberately favourable to fungal growth, means that the experiment represents a relatively high-risk storage scenario rather than conventional oat storage. Thus, the study provides evidence that supplementation with antioxidant-rich ingredients, particularly cinnamon, may limit HT-2 toxin accumulation under conditions favourable to fungal activity, but further studies using a larger number of independent oat samples and more representative storage conditions are required to establish the practical relevance of this approach.

### 3.3. Dehulling and Scouring

The dehulling and polishing (scouring) processes of oat grains have been extensively studied regarding their impact on mycotoxin contamination. These steps are considered critical for improving oat safety and quality before further processing.

Dehulling, which involves the mechanical removal of the outer hull, has been consistently reported to significantly reduce mycotoxin concentrations in oats. Early investigations by Alder et al. [103] demonstrated that dehulling achieved reductions greater than 90% for T-2 toxin, HT-2 toxin, and NIV. Also, Scudamore et al. [92] demonstrated that dehulling achieved reductions greater than 80% for DON, NIV, HT-2, and T-2 toxins. Pettersson et al. [91] similarly observed a mean reduction of 80–95% for T-2 and HT-2 toxins following dehulling (combined with a previous cleaning), reinforcing the effectiveness of the process. The reduction in these type A trichothecenes was lower at lower levels of contamination. Hietaniemi et al. [73] further demonstrated that most of the mycotoxin burden was concentrated in the hull fraction, leading to non-detectable T-2 toxin and HT-2 toxin levels in the groats, while DON and 3-ADON were reduced by 67–91% after sorting and dehulling.

Schwake-Anduschus et al. [80], through laboratory trials, confirmed that dehulling consistently led to significant T-2 toxin and HT-2 toxin reductions across multiple oat cultivars, although variability existed among different agricultural sites, with reductions ranging from 94 to over 100% depending on the initial contamination and grain characteristics. An interesting result from this work is that after dehulling, exclusively T-2 toxin was found at low levels in kernels, indicating that HT-2 toxin is more strictly confined to oat hulls. Additional confirmation comes from Ivanova et al. [104], who assessed the effects of primary oat processing steps, specifically dehulling and pearling (also referred to as scouring), on the distribution and reduction in DON, 3-ADON, T-2 toxin, HT-2 toxin, and their plant-derived conjugates DON-3G, T-2-glucoside, and HT-2–3-glucoside. Their findings demonstrated that dehulling was highly effective in reducing mycotoxin levels, with DON reduced by 94%, 3-ADON by 91%, DON-3G by 83%, T-2 and HT-2 toxins by approximately 94% and 95%, respectively, and T-2-glucoside showing a comparable reduction of 93%. This efficacy is attributed to the strong localisation of these toxins in the hulls, which were removed during dehulling, accounting for roughly 31–43% of the whole grain weight. The findings indicate that fungi possess a limited ability to penetrate the hull and colonise the endosperm or germ. These authors reported that fungal DNA was predominantly located in oat fractions corresponding to the hull, bran, and outer endosperm layers, which aligns with the mycotoxin distribution observed, suggesting that most mycotoxins are not readily translocated from the hull into the oat grain. Following dehulling, the same authors observed that pearling further contributed to the reduction in mycotoxins, albeit to a lesser extent. Dehulling and pearling revealed different distribution patterns for free and modified mycotoxins. Although DON and its related metabolites generally decreased towards the kernel core, the relative proportion of DON-3G was higher in the oat kernel and the outer fraction removed during the initial pearling stage (0–3 min) than in the hull. A similar shift towards the glucosylated form was observed for HT-2–3-glucoside, although conclusions for type A trichothecenes were limited by the small number of samples analysed. The authors suggested that these patterns may reflect plant detoxification through glucosylation but also acknowledged that differences in molecular mobility and transport between parent and modified toxins may contribute to their uneven distribution within the grain. Thus, the relative enrichment of glucosylated forms in edible kernel fractions should not be attributed solely to their site of formation. This differential distribution is relevant to food safety because modified forms retained in edible fractions may contribute to total dietary exposure and may potentially be converted back to their parent toxins during digestion. At laboratory scale, Tittlemier et al. [105] also confirmed that dehulling is highly effective in removing the majority of *Fusarium* mycotoxin mass from oats, with 60–100% being associated with the hull fraction. For most analytes, median concentrations were higher in hulls than in groats, supporting the role of dehulling as a key physical mitigation step. ZEN, NIV, HT-2 toxin, and 3-ADON occurred infrequently and at low concentrations and were only quantifiable in hulls, remaining below the LOQ in groats. The median reduction in DON achieved through dehulling was 87%, while the proportion of mycotoxin mass associated with hulls ranged from 48% for DON-3G to 100% for HT-2 toxin and 3-ADON. Notable exceptions were observed for BEA and ENN A_1_, for which median concentrations appeared higher in groats than in hulls, highlighting a potential food safety concern for compounds less strongly associated with outer grain tissues. Furthermore, consistent with observations by Ivanova et al. [104], the relative DON/DON-3G ratio in processed grains indicated a higher relative abundance of DON-3G, suggesting facilitated redistribution or retention of this modified mycotoxin within inner grain layers. More recently, Gordon et al. [106] provided further evidence of this differential distribution by analysing free and glucosylated *Fusarium* mycotoxins in unprocessed oats, hulls and groats following laboratory dehulling. More than 90% of the mycotoxin load was removed with the hull for most compounds, with reduction factors of 93% for T-2 toxin, 98% for HT-2 toxin, 96% for HT-2-glucoside, 97% for T-2-glucoside, 93% for DON and 92% for ZEN. In contrast, DON-glucoside showed a lower reduction of 77%, supporting previous observations of relatively greater retention of glucosylated DON in inner grain fractions compared with the parent toxin. Importantly, despite these high reduction factors, free and glucosylated T-2 and HT-2 toxins remained detectable in a proportion of the dehulled groats, indicating that substantial physical removal during dehulling does not necessarily result in complete elimination from the edible fraction. As explained in Section 3.1, Dropa et al. [7] reinforced that the most significant decrease in mycotoxins occurred in dehulling and cleaning steps, documenting reductions of approximately 70–80% for ENN B, 75–87% for ENN B_1_, 48–100% for HT-2 toxin, 58–80% for T-2 toxin, and 35–65% for BEA. Meyer et al. [90] examined the effects of grain sorting and both commercial and laboratory dehulling on the reduction in DON, T-2 toxin, and HT-2 toxin in milling oats. Prior to dehulling, oats were sorted into large and thin kernels to optimise impact energy during processing and improve yield. As mentioned in Section 3.1, this classification had significant implications for mycotoxin removal, as thin grains tended to retain higher levels of contamination due to their greater susceptibility to *Fusarium* infection. In commercial-scale dehulling trials, mean DON reductions of 61% and 57% were achieved for large and thin grains, respectively, while the sum of T-2 and HT-2 toxins was reduced by 85% in large grains and 65% in thin grains. Consistent with the commercial-scale trials, laboratory dehulling resulted in average reductions of 84% for T-2 and HT-2 toxins and 72% for DON. Importantly, the reduction in toxin levels was, as seen by Ivanova et al. [104], strongly correlated with the decrease in *F. graminearum*, *F. culmorum*, and *F. langsethiae* DNA, indicating that dehulling effectively removes fungal biomass along with associated mycotoxins. Also, these authors investigated the efficacy of laboratory-scale polishing (scouring) in reducing these mycotoxin levels. Their study demonstrated that removing just 3.2% of the outer layers of oat grains led to a significant average reduction of 32% for DON and 42% for the sum of T-2 and HT-2 toxins. This suggests that a substantial portion of these toxins is concentrated in the outer grain layers (used as bran). However, the remaining 97% of the grain still retained approximately two-thirds of the initial mycotoxin load, indicating that polishing alone does not eliminate contamination. 

A crucial aspect to highlight is the fate of the removed hulls, which are often used as by-products for animal feed in the form of pellets. Several studies showed that these hulls can contain toxin concentrations several times higher than the original oat grains [90,91,92,107,108]. Pettersson et al. [91] observed T-2 and HT-2 toxin enrichment factors of 3- to 6-fold in milling by-products. Similarly, Edwards et al. [107] reported that the concentration of HT-2 and T-2 toxins in the pelleted by-product was on average 4.4 times greater than in the unprocessed oats, with extreme cases showing a shift from 4528 μg/kg in raw oats to 29,700 μg/kg in the pelleted co-product. Scudamore et al. [92] expanded on this by demonstrating that the concentration of DON, NIV, HT-2 toxin, and T-2 toxin was consistently far higher in the pellet than in raw oats or oat flakes. Maximum pellet concentrations exceeded 4000 µg/kg for DON, 600 µg/kg for NIV, 20,000 µg/kg for HT-2 toxin, and 6000 µg/kg for T-2 toxin. Enrichment factors ranged from 1.7- to 15-fold, and the highest values tended to occur when initial contamination in the raw oats was already elevated. Additionally, related type A trichothecenes such as NEO and T-2 triol were also sometimes detected in this co-product, reflecting the concentration of toxins in the outermost grain layers. Meyer et al. [90] corroborated these findings, showing that husks could contain nearly 2.5 times the initial DON, T-2 toxin, and HT-2 toxin levels present in milling oats. These studies consistently show that *Fusarium* mycotoxins are concentrated in the hull fraction, which is consistent with the detection of relatively high DON and ZEN contamination in oat bran lots, where 17% of samples tested positive for each toxin, with maximum concentrations of 276 µg/kg for DON and 25 µg/kg for ZEN [70]. While the effectiveness of primary processing in protecting the human food chain is evident, a significant downside is also revealed: the potential risk posed to livestock when these mycotoxin-rich by-products are used as feed. Without proper monitoring and mitigation strategies, such use could lead to unintended exposure in animals, particularly in sensitive species. Therefore, strict quality control and risk assessment protocols are essential to ensure the safe use of these oat-derived co-products in animal nutrition.

Collectively, these findings demonstrate that dehulling is a critical control step in oat processing for mitigating mycotoxin contamination. However, they also highlight important safety considerations for by-products, which may retain substantial toxin levels. While dehulling remains the most effective intervention, pearling can provide additional, albeit more limited, mitigation.

### 3.4. Steaming and Kilning

Steaming and kilning are essential thermal processing steps applied early in the oat supply chain, primarily during the production of oat groats and flakes. Their main technological objectives are to inactivate endogenous lipase enzymes, which are responsible for rancidity, stabilise the oat grain, and enhance flavour and aroma development [76]. Steaming typically involves exposing the oats to saturated steam at high temperature, often under pressure, while kilning consists of dry-heat treatment applied to reduce moisture content and complete enzyme deactivation [77]. These processes are not primarily designed for mycotoxin control; however, their thermal nature raises the question of whether they can contribute to reducing or altering mycotoxin content.

In a detailed investigation of commercial oat processing lines in the UK, Scudamore et al. [92] followed the fate of *Fusarium* toxins throughout the full production chain. They observed that kilning had only a minor impact on T-2 toxin, HT-2 toxin, NIV, and DON levels, with most reduction occurring during dehulling and physical sorting, as mentioned above. Commercial kilning may involve process temperatures of approximately 100–110 °C for 1–2 h [92], although these conditions do not necessarily correspond to the temperature reached by the oat grains throughout the entire treatment. The limited effect of kilning on mycotoxin concentrations is consistent with the relatively high thermal stability of several *Fusarium* mycotoxins. Kabak [109] reported melting points of 151–153 °C for DON and 164–165 °C for ZEN, although these values should not be interpreted as direct thresholds for thermal degradation. DON was reported to remain stable during heating at 100–120 °C at pH 4–7, whereas reductions of up to 52% were observed following treatment with superheated steam at 185 °C for 6 min. ZEN also showed considerable thermal stability, with no complete degradation reported even after heating at 120 °C for 4 h [109].

More recent work by Dropa et al. [7] examined mycotoxin dynamics during the industrial production of oat flakes, including kilning and steaming, applied at 90–100 °C for 10–20 min. The authors reported that these thermal steps contributed to some additional reduction in *Fusarium* mycotoxins; however, their impact was modest when compared with upstream operations such as cleaning and dehulling. Across the overall process, total reductions in T-2 toxin, HT-2 toxin, ENNs and BEA ranged from 32% to 100%, with higher apparent reductions generally observed for HT-2 toxin and ENNs than for T-2 toxin and BEA. Given the relatively mild thermal conditions applied and the known heat stability of these mycotoxins, the observed decreases are largely attributed to the physical removal of contaminated outer grain layers rather than to direct thermal degradation during kilning and steaming.

The study by Tittlemier et al. [105] investigated the influence of laboratory-scale steaming (100 °C) and kilning (102 °C, 45 min) on oats naturally contaminated with multiple *Fusarium* mycotoxins. DON and DON-3G concentrations were consistently lower in steamed and kilned groats as opposed to untreated groats. DON concentrations decreased in six out of seven detected cases (mean reduction of 27%), while DON-3-G levels fell in four out of six instances (mean reduction of 20%) following steaming and kilning. They suggested that this slight reduction in type B trichothecenes was due to the combination of heat and steam. 

Complementing these findings, Kuchenbuch et al. [110] evaluated the effect of pressure cooking in the preparation of flaked oats from artificially contaminated samples. This study reported that pressure cooking led to only minor changes in T-2 toxin and HT-2 toxin concentrations (approx. 17% for T-2 toxin and 18% for HT-2 toxin), similar to what Tittlemier et al. [105] observed for DON and DON-3G. Consequently, pressure cooking is insufficient to ensure effective detoxification, resulting in only limited reductions in these mycotoxins.

### 3.5. Cutting and Flaking

The key steps of cutting and flaking are responsible for transforming kilned groats into rolled oats or instant oat products. In these steps, groats are often cut using steel blades and subsequently passed through large flaking rollers to produce flat, thin oat flakes. These operations are carried out at moderate temperatures, and although not specifically designed for mycotoxin mitigation, they may influence mycotoxin concentrations indirectly through mechanical redistribution or physical loss.

One of the most detailed investigations on this topic was conducted by Dropa et al. [7], who monitored the evolution of mycotoxin concentrations throughout the industrial production of oat flakes. While cutting and flaking contributed to additional reductions in T-2 toxin, HT-2 toxin, BEA, and ENNs, their impact was notably smaller compared to earlier processing stages. For instance, the concentration of HT-2 toxin in oats after cleaning and dehulling was 10.2 µg/kg, whereas in all subsequent processing steps it remained consistently below 5 µg/kg. In the case of T-2 toxin, oats prior to steaming and cleaning contained 4.3 µg/kg, which decreased slightly to 4.2 µg/kg and 2.9 µg/kg in different batches. Following flaking, final levels in oat flakes were further reduced to 3.0 µg/kg and 2.1 µg/kg, respectively. These modest decreases are consistent with the low thermal intensity and mechanical nature of the cutting and flaking operations, and they underscore the greater significance of earlier decontamination steps such as cleaning, dehulling, and steaming in reducing mycotoxins in oats.

Scudamore et al. [108] reported similar observations in an industrial-scale study conducted in the UK. Oat flake production was shown to result in significant reductions in DON, NIV, T-2 toxin, and HT-2 toxin levels when compared to raw oat grains, but the changes were largely attributed to cleaning and dehulling. The author emphasised that the mechanical nature of flaking, along with the possible removal of fine material during rolling and sieving, could account for minor additional decreases in toxin concentration. However, no evidence of chemical degradation was found during the flaking and cutting stage itself. 

A broader view on the distribution of mycotoxins across oat processing streams was provided by Pettersson et al. [111], who analysed raw oats (*n* = 243) and oat flakes (*n* = 529) from 11 European mills. They reported that mean T-2 toxin and HT-2 toxin levels in oat flakes were reduced by approximately 82% compared to the raw oats. Although the authors did not quantify the specific effect of cutting and flaking, the data suggest that these steps contribute to the final clean-up of the product.

A more advanced scenario was described by Kuchenbuch et al. [110], who investigated a modern approach to flaking that incorporates pressure-cooking, extrusion, and roasting prior to flaking. Their study revealed that thermal degradation of T-2 and HT-2 toxins occurred progressively across the process stages. After pressure-cooking, both toxins were reduced by approximately 17% (as explained in the previous section). Extrusion cooking did not further affect HT-2 toxin but led to a slight additional reduction in T-2 toxin, resulting in a total degradation of 21% after both thermal steps. A final roasting step at 300 °C for 2 min enhanced toxin degradation significantly: T-2 toxin was reduced by 32% and HT-2 toxin by 24%. These findings confirm that T-2 toxin is more thermolabile than HT-2 toxin, and that high-temperature roasting contributes substantially to toxin reduction. The authors also noted that this pattern aligns with their biscuit-making results and is consistent with findings from other studies showing greater heat sensitivity of T-2 toxin compared to HT-2 toxin [112]. However, it is important to note that these reductions are not attributable to flaking per se, but rather to the preceding extrusion-cooking and subsequent thermal steps embedded in modern cereal flake manufacturing.

### 3.6. Oat Flour Processing

The transformation of whole oats into oat flour is a key stage in the oat processing chain and central to the production of various oat-based food products [113]. However, this process can also play a critical role in shaping the distribution and potential mitigation of mycotoxins. Recent studies have explored the fate of these toxins under different flour processing methods, including milling, extrusion cooking, roasting, enzymatic treatment, and novel non-thermal approaches such as cold plasma.

#### 3.6.1. Milling and Redistribution of Toxins

While milling does not chemically degrade *Fusarium* mycotoxins, it significantly affects their physical redistribution among oat fractions. In a regional survey, Pettersson et al. [91] showed that T-2 and HT-2 toxins tend to concentrate in by-products such as screenings, bran, and outer layers. However, refined oat flour generally contains lower levels. This finding illustrates how mechanical processing primarily redistributes mycotoxins among milling fractions rather than eliminating them, highlighting the importance of fraction management to limit contamination in edible products.

Further insights were provided by Tittlemier et al. [105], who evaluated the impact of grinding and sample preparation on the analysis of *Fusarium* mycotoxins in oats. Rather than focusing on industrial fractionation, this study demonstrated how grinding-induced changes in particle size distribution can influence measured mycotoxin concentrations. Grinding generated particles of different sizes that did not contain the same proportion of mycotoxins, and the distribution of DON, DON-3G, NIV, ZEN and ENNs across particle size fractions deviated from the distribution of sample mass, indicating that these toxins are not homogeneously distributed within ground oat material. This behaviour was attributed to uneven localisation of mycotoxins within the oat grain and/or differences in extractability from particles of different sizes. The authors further demonstrated that the type of grinder and grinding conditions affected sample homogeneity, with finer grinding achieved using rotor-beater mills producing more homogeneous material, which is critical for reliable mycotoxin analysis. Consequently, variations in particle size distribution introduced during grinding may alter measured mycotoxin concentrations without changing the total toxin content. In contrast, when finely ground oats were divided into analytical test portions, the use of four different division methods (manual scooping, rotary division and two gravity-fed dividers) did not result in practical differences in particle size distribution, indicating that sample division, under these conditions, is unlikely to bias *Fusarium* mycotoxin analysis. 

In conclusion, these studies show that milling influences mycotoxin outcomes through physical redistribution at both industrial and analytical scales, rather than through detoxification.

#### 3.6.2. Extrusion Cooking

Among available thermal treatments, extrusion cooking has shown promising potential for the reduction in T-2 and HT-2 toxins in oat flour. Schmidt et al. [112] conducted a detailed investigation using both fortified and naturally contaminated oat flours, under laboratory and industrial-scale extrusion conditions. In fortified samples, reductions of up to 60% for T-2 toxin and 47% for HT-2 toxin were observed. In contrast, extrusion of naturally contaminated flours yielded lower reductions, 35% and 22%, respectively, likely due to matrix binding and the presence of modified toxin forms. Key processing parameters affecting degradation included moisture content, mechanical shear, and to a lesser extent, temperature. Critically, the study examined not only toxin loss but also the nature of the degradation. Several known degradation products and metabolites of T-2 toxin were monitored, including NEO, T-2 triol, T-2 tetraol, and three additional degradation products identified in earlier thermal model studies [114]. However, the results showed that only trace amounts of these compounds could be detected. Conversion of T-2 toxin into HT-2 toxin accounted for less than 5% of the total T-2 toxin loss, while T-2 triol and NEO were found at levels corresponding to less than 0.1% of the original T-2 toxin content. The most prominent thermal degradation product, referred to as “degradation product #1”, was only detectable in samples extruded at 200 °C, representing up to 3% of the initial T-2 toxin. Other expected products (e.g., T-2 tetraol, degradation products #2 and #3) were not detected at all. These findings strongly suggest that the majority of T-2 and HT-2 toxins degradation during extrusion does not result in known or easily identifiable breakdown products. Instead, the authors hypothesise that a significant portion of the toxins may be transformed into yet unknown degradation compounds, or possibly into matrix-bound forms that escape conventional detection methods. 

Expanding on this, Pleadin et al. [115] reported even greater reductions in naturally contaminated cereals processed via extrusion, with T-2 toxin and HT-2 toxin reductions ranging from 73% to 100%. Also, Pleadin et al. [116] observed a reduction between 73 and 87% for DON and between 58 and 100% for ZEN. The authors attributed this high efficacy to the intense thermal and mechanical stress during extrusion, which contrasts with conventional cooking methods that resulted in negligible reductions (<10%). Importantly, they stressed that extrusion parameters such as temperature profile, pressure, and residence time must be carefully calibrated to maximise toxin degradation without compromising product quality. These findings confirm extrusion cooking as one of the most effective and scalable mitigation strategies currently available for oat flour products. 

#### 3.6.3. Roasting and Enzymatic Treatment of Oat Flour

In the context of cereal-based infant food production, oat flour often undergoes specific processing steps such as roasting and enzymatic hydrolysis to enhance flavour, solubility, and digestibility [113]. Pascari et al. [79] investigated the impact of these treatments on a range of *Fusarium* mycotoxins, under conditions mimicking industrial baby food production. The roasting process, conducted at 105 °C and 120 °C for up to 40 min, did not significantly alter the concentrations of DON, ZEN, 15-ADON, T-2 and HT-2 toxins, indicating their high thermal stability under the mild temperatures typically applied in this process. Similarly, during enzymatic hydrolysis using α-amylase and glucoamylase, no statistically significant changes in these five mycotoxins were observed, regardless of enzymatic concentration or treatment duration. These findings suggest that neither roasting nor enzymatic treatment, as applied in the manufacture of infant cereals, is effective at reducing *Fusarium* mycotoxins in oat flour. 

#### 3.6.4. Innovative Decontamination: Cold Plasma

A promising alternative to thermal methods is cold plasma treatment, which enables non-thermal detoxification through the generation of reactive gas species. Kiš et al. [117] tested the efficacy of low-pressure dielectric barrier discharge plasma in reducing T-2 and HT-2 toxins in oat flour. Using different gases, they found that nitrogen plasma was the most effective, achieving reductions of 43% for T-2 toxin and 39% for HT-2 toxin, in a time-dependent manner. The mechanism involves oxidative degradation by reactive oxygen and nitrogen species, which can break down toxin molecules without the application of heat. As such, cold plasma holds potential for preserving the nutritional and functional properties of oat flour, though its use remains at pilot scale and requires further development for industrial implementation.

### 3.7. Oat Bakery Products

Bakery processing can play a critical role in determining the fate of *Fusarium* mycotoxins in oat-based products [110]. Recent studies have investigated the extent to which these toxins degrade under different baking conditions and formulations, providing insight into both industrial and domestic processing outcomes.

Schwake-Anduschus et al. [80] evaluated the stability of T-2 and HT-2 toxins during bread production using a formulation containing 20% oatmeal and 80% wheat flour. Their results showed that both toxins were relatively stable during standard baking at 220 °C for 40 min, with an average degradation of only 23%. This modest reduction aligns with the known thermostability of these toxins. Interestingly, HT-2 toxin levels were consistently higher than those of T-2 toxin in raw oats, and this proportion was maintained post-baking, suggesting limited conversion or degradation pathways during the baking process. The stability observed in this bread matrix, which includes yeast fermentation, implies that microbial activity has a limited effect on toxin degradation in the absence of targeted enzymatic action.

Moreover, Kuchenbuch et al. [110] explored the thermal stability of T-2 and HT-2 toxins in non-yeasted oat bakery products such as biscuits and crunchy muesli, using controlled laboratory-scale experiments with artificially spiked oats. The degradation of toxins was influenced by several factors: product composition, baking temperature and time, water content, and sugar type. Biscuit-making led to a partial degradation of both toxins, with T-2 toxin reductions of up to 45% and HT-2 toxin reductions of up to 20%. The extent of degradation was greater at lower water content and longer baking times, highlighting the importance of moisture in modulating heat transfer and chemical reactions. Higher initial moisture content delayed internal temperature rise, reducing thermal load on the toxins. Moreover, the type of sugar used also had an effect: surprisingly, sucrose contributed more to degradation than glucose, possibly due to different reaction pathways under heat. However, in crunchy muesli production, which involves lower baking temperatures (150–170 °C) and a dry-air roasting system, degradation was minimal, with reductions of only 8–19%, depending on temperature and toxin type. This limited effect is consistent with the lower thermal energy applied and the brief exposure times typical of muesli processing.

Regarding bakery products made from other cereals, particularly wheat, the behaviour of DON, ZEN, and several of their modified forms during baking has been more extensively investigated [46,47,118,119]. These studies consistently indicate that DON and ZEN may undergo partial reduction during baking, with the extent of degradation strongly dependent on temperature, baking time, moisture content, and product formulation, and with highly variable outcomes reported across different products and processing conditions. In addition, baking has been shown to affect the occurrence of modified forms of DON and ZEN, including both their partial degradation and, in some cases, their release or formation under specific thermal conditions. However, despite the relevance of DON and ZEN as major *Fusarium* mycotoxins in oats, just one study has evaluated their fate, or that of their modified forms, during the production of oat-based bakery products [120]. Their results also indicated that mycotoxin reduction in oat biscuits depended significantly on baking temperature and duration. ZEN exhibited higher thermostability than DON-3G, and DON-3G was more thermostable than DON. Under harsh conditions, 15-ADON decreased while DOM-1 increased. Moreover, other metabolites such as isoDON and norDONs emerged during baking. ZEN, α-zearalenol-14-S (α-ZEL-14-S), and β-zearalenol-14-S (β-ZEL-14-S) showed increased levels during the initial baking phases due to the release of hidden mycotoxins, raising safety concerns. Overall, the final edible biscuits for each temperature exhibited similar DON and DON-3G concentrations, with higher ZEN levels than initially, not reducing the mycotoxin risk. 

### 3.8. Oat Porridge Preparation

The preparation of oat porridge (also called oatmeal in several countries) represents a key thermal processing step in the consumption of oat-based products, particularly for infants, children, and health-conscious populations [121]. 

In a detailed study by Schwake-Anduschus et al. [80], the effect of porridge preparation on T-2 and HT-2 toxins was investigated using naturally contaminated oat material. Porridge was prepared from whole oatmeal obtained from cleaned, but non-dehulled, oats by mixing 20 g of meal with 100 mL of water and heating from 30 °C to 96 °C over 22 min, followed by 1 min at the maximum temperature, using a Micro Visco-Amylo-Graph to ensure adequate mixing during preparation. Five oat samples with different initial T-2 and HT-2 toxin concentrations were evaluated, with three replicate porridge preparations per sample. Toxin concentrations in the resulting porridges showed considerable variability, with mean changes ranging from a 40% reduction to a 46% increase relative to the theoretical concentrations expected in the absence of toxin alteration. Given this variability, the authors could not establish a significant effect of porridge preparation on T-2 and HT-2 toxin concentrations.

The experimental design and sampling procedure are important considerations when interpreting these findings. Analytical variability was considered unlikely to fully explain the observed differences, since matrix effects were evaluated using matrix-matched calibration and satisfactory recoveries were obtained for both toxins in the porridge matrix. However, the source of the large variability among replicate porridge preparations remained unresolved. In particular, the study does not provide sufficient detail to determine whether the three replicate preparations for each oat sample were obtained from a single homogenised meal or from separately processed subsamples. Consequently, a contribution from subsampling or sample heterogeneity cannot be excluded. The relatively small number of naturally contaminated oat samples, together with the substantial variability among replicate preparations, further limits the precision with which the effect of cooking can be quantified.

The representativeness of the starting material should also be considered. Cleaned but non-dehulled oats were deliberately used because dehulling markedly reduced T-2 and HT-2 toxin concentrations, potentially resulting in levels too close to the analytical detection limits for the cooking experiment. Although this approach facilitated the assessment of toxin behaviour during heating, it does not fully represent the oat material normally used for human consumption. Therefore, direct extrapolation of the observed changes to conventional domestic or commercial porridge preparation should be made with caution. These experimental and sampling limitations prevent a precise quantification of the effect of porridge preparation on T-2 and HT-2 toxin concentrations. Nevertheless, the absence of evidence for a consistent reduction remains relevant from an exposure perspective, particularly for young children, who may consume relatively high amounts of oat porridge in relation to their body weight.

More recently, Gordon et al. [106] provided complementary evidence on the presence and bioaccessibility of type A trichothecenes in oat porridge. Two naturally contaminated commercial porridge oat samples were cooked by boiling and subsequently subjected to an in vitro gastrointestinal digestion model. T-2 toxin, HT-2 toxin and HT-2-glucoside were quantifiable in the prepared porridges, whereas DON, NIV, DAS, ZEN and their glucosylated forms were below the LOQ in these samples. Following simulated gastrointestinal digestion, T-2 and HT-2 were highly bioaccessible, and HT-2-glucoside was particularly efficiently released from the porridge matrix. When expressed as T-2 equivalents, the combined bioaccessibility of T-2, HT-2, and HT-2-glucoside across the upper and lower gastrointestinal compartments reached 73–82%. Although the study was not designed to quantify changes in mycotoxin concentrations specifically attributable to porridge cooking, these findings demonstrate that free and glucosylated type A trichothecenes can be present in prepared oat porridge in highly bioaccessible forms and therefore remain relevant to dietary exposure.

In a broader industrial context, a large-scale survey by Pettersson et al. [111] analysed T-2 toxin and HT-2 toxin levels in industrially produced oat products across 11 European mills between 2005 and 2009. Among 105 oatmeal samples, the incidence of contamination (T-2 and HT-2 toxins > 5 µg/kg) was 34%, with mean and median values of 11 µg/kg and 5 µg/kg, respectively. The lower levels found in oatmeal compared with raw oats (mean: 94 µg/kg) are consistent with the substantial reductions achieved during industrial cleaning and dehulling, which were reported to reduce toxin levels by 82–88%.

Available evidence therefore does not demonstrate a consistent reduction in T-2 and HT-2 toxins during porridge preparation, suggesting that conventional cooking should not be relied upon as an effective mitigation step. The low toxin concentrations reported in commercial oatmeal appear instead to reflect reductions achieved during earlier industrial processing, particularly cleaning and dehulling, rather than during porridge preparation itself. Recent evidence further indicates that free and glucosylated type A trichothecenes can be present in prepared oat porridge and may be highly bioaccessible, reinforcing their relevance to dietary exposure. However, direct quantitative evidence on processing-induced changes during porridge preparation remains very limited, particularly for modified forms and for *Fusarium* mycotoxins other than T-2 and HT-2. Further studies comparing mycotoxin concentrations and mass balances before and after porridge preparation under representative domestic conditions are therefore needed to determine whether cooking causes degradation, transformation, release from the matrix, or primarily persistence of these compounds.

### 3.9. Oat-Based Beverages (OBBs)

OBBs are increasingly consumed as plant-based alternatives to dairy products [122]. However, despite their growing market relevance, information on the occurrence and behaviour of *Fusarium* mycotoxins in these products remains limited. Although OBBs are manufactured from oat flour or flakes through enzymatic liquefaction and subsequent thermal treatments, most available studies have focused on the presence of mycotoxins in final commercial beverages rather than on their behaviour during processing.

Data on mycotoxin occurrence in OBBs indicate generally low contamination levels, albeit with substantial variability between studies. In a survey of OBBs, Miró-Abella et al. [68] reported DON concentrations consistently below 15 µg/L, along with low-level detection or non-detection of other *Fusarium* mycotoxins, such as ZEN, T-2 toxin, HT-2 toxin, and FUMs. In contrast, Hamed et al. [123] detected DON in three OBBs at considerably higher concentrations (191–270 µg/L), demonstrating that significant carry-over from contaminated raw cereals to beverages may occur. These contrasting findings highlight the strong influence of raw material quality and upstream processing on final mycotoxin levels in OBBs.

A broader survey by Pavlenko et al. [124] investigated the occurrence of 22 mycotoxins in commercial OBBs. Overall, mycotoxin concentrations were low, typically below 15 µg/L. FUMs, NIV, 3-ADON, and 15-ADON were not detected, whereas ENNs (particularly ENN B and ENN B_1_), DON, BEA, HT-2, and T-2 toxins were the most frequently observed compounds. The highest concentrations were reported for DON and ENN B_1_, reaching approximately 9 and 3.2 µg/L, respectively. The same study further evaluated the impact of consumer-level thermal treatments, including boiling and coffee-machine preparation, on mycotoxin concentrations in OBBs. Apparent reductions were observed in some cases, with boiling resulting in decreases of approximately 8–18% and coffee-machine heating leading to reductions of up to 100% for certain toxins. However, the authors emphasised that the very low initial contamination levels complicate interpretation, as small analytical variations or matrix effects may disproportionately influence calculated reductions. Although localised steam temperatures of up to 130 °C were suggested as a possible contributing factor, the relevance of these treatments for mycotoxin mitigation in OBBs remains uncertain.

Teixido-Orries et al. [125] conducted a detailed process-based study using oat flour with DON, providing a valuable insight into how mycotoxins may transfer through OBB production stages. The process involved mixing oat flour with water, enzymatic liquefaction using α-amylase and glucoamylase at 65 °C, centrifugation, and thermal sterilisation at 121 °C for 15 min. Throughout this workflow, DON was found to transfer efficiently from the flour to the final beverage (66–78%), with no significant reduction during thermal sterilisation. The study also showed that formulation factors (such as oat content) and enzymatic treatment strongly influenced final DON concentrations. Although DON was the only mycotoxin evaluated, the findings suggest that oat beverage processing does not inherently eliminate *Fusarium* toxins, and that a substantial portion of the initial contamination can be retained in the final product. 

Notably, OBBs are not subject to specific maximum limits for mycotoxins under current EU legislation, despite their rapidly increasing consumption and nutritional relevance. Moreover, no studies have specifically investigated fermented products derived from OBBs, such as oat “yoghurts”, in which additional changes in mycotoxin concentrations or forms may occur during fermentation. Taken together, these gaps highlight the need for process-oriented studies to elucidate the fate and transformation of *Fusarium* mycotoxins throughout the production of OBBs and their derived products.

In addition to the liquid fraction, OBB manufacture generates solid by-products such as oat press cake, obtained after enzymatic liquefaction and solid–liquid separation. This fraction may retain a substantial proportion of the original mycotoxin load and therefore represents a relevant matrix for evaluating downstream mitigation strategies. In this context, Bartkiene et al. [126] investigated alternative processing approaches applied to DON-contaminated oat press cake. Low-frequency ultrasonication was found to be ineffective, while fermentation with a *Lactobacillus casei* strain not only failed to reduce DON contamination but also resulted in increased concentrations of DON and 15-ADON, highlighting the potential for unfavourable transformations during biological treatments.

### 3.10. Experimental Thermal Treatments of Oat Grains

In addition to the thermal operations included in conventional oat processing, several studies have investigated the thermal stability of *Fusarium* mycotoxins in whole oat grains under experimental conditions that do not necessarily represent standard industrial practices. Although their direct applicability to commercial oat processing is limited, these studies provide valuable insight into the response of mycotoxins to different combinations of temperature, moisture, and exposure time.

Pleadin et al. [115] evaluated the effectiveness of cooking in boiling water (96 °C) as a mitigation method for T-2 toxin and HT-2 toxin in naturally contaminated oat grains. Cooking was performed for 10, 20, and 30 min. The results revealed that cooking had a very limited effect, with total T-2 toxin and HT-2 toxin reductions not exceeding 9%, and individual reductions of up to 15% for T-2 toxin and 8% for HT-2 toxin. Increasing the cooking time beyond 10 min did not significantly improve the outcome. Similar behaviour has also been reported for DON and ZEN, which exhibit high thermal stability during cooking in boiling water, with no significant additional reductions observed when cooking time is extended from 10 to 30 min [116]. These findings underscore the thermal stability of *Fusarium* mycotoxins under moist, moderate heat conditions. The process conditions applied cannot achieve meaningful detoxification. Moreover, as boiling is not applied at the grain level in the oat processing industry, its practical relevance remains minimal. 

In contrast to cooking, roasting of whole oat grains was shown to be a more promising thermal approach. Pleadin et al. [115] tested roasting at 180 °C, 200 °C, and 220 °C for 30 min. The total reduction in T-2 toxin and HT-2 toxin ranged from 33% at 180 °C to 54% at 220 °C, with T-2 toxin exhibiting greater thermal sensitivity than HT-2 toxin. Notably, T-2 toxin was eliminated in oats roasted at 220 °C, while HT-2 toxin was reduced by 43%. A similar temperature-dependent response was observed for DON and ZEN, whose reductions increased progressively with roasting severity, reaching up to 36–40% for DON and 33–55% for ZEN at 220 °C [116]. Compared with cooking, roasting resulted in substantially higher reductions in *Fusarium* mycotoxins, consistent with the established importance of processing temperature and exposure time in driving mycotoxin degradation. Although these results demonstrate the potential of dry heat for toxin reduction, roasting is not a standard practice applied to raw oat grains in milling or primary processing environments. It is more typical of final-product manufacturing (e.g., for toasted oats or granola) or flour treatment, where quality attributes such as flavour, colour and texture are of primary concern. Therefore, while roasting can effectively reduce toxin levels under controlled laboratory conditions, its applicability at the grain level in industrial oat processing is very limited.

### 3.11. Impact of Oat Processing on Mycotoxin Co-Occurrence and Mixture Composition 

Oats are commonly contaminated by multiple *Fusarium* mycotoxins rather than by individual compounds in isolation, including the simultaneous occurrence of parent toxins, their modified forms and, in some cases, emerging mycotoxins. Processing therefore affects not only the concentration of individual mycotoxins but also the composition of the contaminant profile reaching the final product. This distinction is important because the different compounds present in a mixture are not necessarily removed, redistributed, or transformed to the same extent. Consequently, a substantial reduction in one parent mycotoxin does not necessarily imply an equivalent reduction in the total toxicologically relevant group or in the overall mycotoxin burden. The processing data reviewed in the preceding sections provide particularly clear examples for the DON and T-2/HT-2 groups, for which EFSA has established group-based health guidance values that incorporate relevant modified forms [38,51].

For DON, the group TDI of 1 µg/kg body weight/day applies to the sum of DON, 3-ADON, 15-ADON and DON-3G [51]. Consideration of these compounds as a group changes the interpretation of some of the processing reductions reported for oats. The clearest evidence arises from dehulling, where DON and its modified forms do not appear to be removed proportionally. Ivanova et al. [104] reported reductions of 94% for DON and 91% for 3-ADON, compared with 83% for DON-3G, and observed a greater relative contribution of DON-3G in inner grain fractions after removal of the hull. Tittlemier et al. [105] similarly found that only 48% of the DON-3G mass was associated with the hull, whereas the median reduction in DON was 87%. In the same study, 3-ADON occurred infrequently and at low concentrations and was quantifiable only in hulls, with the quantifiable mass associated with this fraction, although the limited occurrence of 3-ADON restricts the strength of conclusions regarding its partitioning. More recently, Gordon et al. [106] again observed preferential removal of DON relative to its glucosylated form, with reductions of 93% and 77%, respectively. Collectively, these findings indicate that dehulling preferentially transfers DON towards the removed outer fraction, while the more limited available evidence suggests a similar tendency for 3-ADON. In contrast, a substantially greater proportion of DON-3G may remain associated with the edible grain. Consequently, dehulling not only reduces the absolute DON group burden but also changes its composition, increasing the relative importance of DON-3G in the retained fraction. This distinction is directly relevant to interpretation of the group TDI, because DON-3G contributes to the same health-based guidance value as DON and its acetylated forms. Importantly, equivalent oat-specific dehulling data are lacking for 15-ADON. Consequently, despite the inclusion of DON, 3-ADON, 15-ADON and DON-3G within the same group TDI, current evidence does not allow the effect of dehulling on the complete DON group to be quantitatively established. Nevertheless, the differential behaviour observed for DON and DON-3G demonstrates that percentage reduction in DON alone cannot be assumed to represent an equivalent proportional reduction in the toxicologically relevant DON group.

The composition of the DON group may be further modified during subsequent processing because its individual members can differ in stability and may undergo interconversion, release from the matrix, or formation of degradation products. This is illustrated by oat-biscuit processing, where DON-3G showed greater thermal stability than DON, 15-ADON decreased under severe processing conditions, and additional DON-related products, including DOM-1, isoDON, and norDONs, were detected [120]. Therefore, processing may progressively change the composition of the DON-related profile, resulting in a chemically different mixture of parent, modified, and transformation products. Importantly, disappearance of DON from the analytically measured profile cannot automatically be equated with an equivalent decrease in toxicologically relevant exposure when other members of the group persist or when the toxicological relevance of newly formed products remains unresolved. This distinction also illustrates why complete group-based mass balances would be more informative than reduction factors calculated only for the parent compound.

A similar group-based interpretation is required for T-2 and HT-2 toxins. EFSA established a group TDI of 0.02 µg/kg body weight/day for T-2 and HT-2 toxins and relevant modified forms [38]. In contrast to the DON/DON-3G pattern, available evidence suggests that dehulling can remove several members of the T-2/HT-2 group with similarly high efficiency. Ivanova et al. [104] reported reductions of approximately 94–95% for T-2 and HT-2 toxins and 93% for T-2-glucoside, while Gordon et al. [106] observed reductions of 93% for T-2, 98% for HT-2, 97% for T-2-glucoside, and 96% for HT-2-glucoside. Thus, unlike the preferential retention of DON-3G observed within the DON group, there is currently no comparable indication that dehulling strongly enriches one of the measured T-2/HT-2 forms in the edible fraction. From a group-TDI perspective, this relatively uniform removal is important because dehulling appears to reduce parent T-2 and HT-2 toxins together with their measured glucosylated forms, rather than selectively reducing only one component of the toxicologically relevant group. Nevertheless, high group-wide removal during dehulling should not be interpreted as complete elimination, since Gordon et al. [106] still detected free and glucosylated T-2/HT-2 forms in a proportion of the resulting groats. Moreover, transfer to hulls and other outer fractions represents redistribution rather than elimination from the wider food and feed chain, particularly when these fractions are subsequently valorised. In such cases, dehulling reduces exposure through the primary edible oat fraction but may redistribute part of the T-2/HT-2 group burden to other points of the food or feed chain.

The relative composition of the T-2/HT-2 group can subsequently change during thermal processing because T-2 and HT-2 toxins do not always show equivalent stability. During combined pressure-cooking, extrusion, and roasting, Kuchenbuch et al. [110] observed progressively different reductions in T-2 and HT-2 toxins, while Schmidt et al. [112] also demonstrated differences in their degradation during extrusion. Under more severe roasting conditions, T-2 was completely removed whereas HT-2 was reduced by only 43% [116]. These compound-specific responses mean that processing can change the T-2:HT-2 ratio even when the concentration of both toxins decreases. From a risk-assessment perspective, however, such redistribution within the group does not necessarily represent a proportional reduction in group exposure because T-2 and HT-2 contribute jointly to the same health-based guidance value. The same principle extends to relevant glucosylated forms: their persistence becomes particularly important when processing preferentially reduces one of the parent toxins.

Recent evidence strengthens the relevance of this interpretation for actual oat foods. Gordon et al. [106] found frequent multi-mycotoxin contamination in commercial oat products and demonstrated the simultaneous presence of T-2, HT-2 and HT-2-glucoside in prepared oat porridge. Moreover, T-2 and HT-2 toxins were highly bioaccessible following simulated gastrointestinal digestion, while HT-2-glucoside was also efficiently released from the porridge matrix. Expressing these compounds as T-2 equivalents increased the estimated group exposure compared with consideration of the free toxins alone. These observations show that modified forms remaining after processing are not merely an analytical consideration but may contribute to the exposure associated with the consumed product. They also reinforce the need to distinguish between removal from a processing stream and reduction in the toxicologically relevant burden reaching the gastrointestinal tract.

An additional complication is that the measured composition of the T-2/HT-2 group may depend on the analytical accessibility of modified forms. Dolezalova et al. [127] demonstrated the presence of non-extractable T-2/HT-2 glycosides in oat products that became quantifiable following enzymatic treatment, indicating that conventional extraction may not fully capture matrix-associated modified forms. This has direct implications for interpretation within the group-TDI framework because relevant glucosylated forms are included in the T-2/HT-2 group health-based guidance value [38]. Therefore, failure to quantify matrix-associated glycosides may lead to underestimation of the analytically determined T-2/HT-2 group concentration and, consequently, of dietary exposure relative to the group TDI. However, the enzymatic treatment used by Dolezalova et al. [127] represents an analytical approach to release non-extractable forms and does not demonstrate that the entire released fraction becomes bioavailable during human digestion. These findings nevertheless show that assessment of the T-2/HT-2 group depends not only on the compounds included in the analytical method but also on their extractability from the food matrix. Consequently, apparent changes in mycotoxin concentrations during processing may reflect physical removal or chemical transformation, but also changes in matrix association and analytical recoverability, which should be considered when establishing group-based mass balances and evaluating processing-related changes in exposure.

The implications of processing for mixture composition extend beyond compounds currently covered by group TDIs. Naturally contaminated oats may simultaneously contain DON-related compounds, T-2 and HT-2 toxins, NIV, ZEN, and emerging mycotoxins such as ENNs and BEA. The evidence reviewed here indicates that a single processing operation can affect these compounds very differently. During cleaning, sorting, dehulling, and scouring, for example, most trichothecenes are preferentially removed from the edible oat fraction and redistributed into processing fractions such as foreign material, rejected grains, hulls, and millfeed. However, this pattern is not consistent across all *Fusarium* mycotoxins. Tittlemier et al. [105] observed higher median concentrations of BEA and ENN A_1_ in groats than in hulls, while Dropa et al. [7] showed that the extent of removal varied considerably among mycotoxins, with BEA being less effectively reduced (35–65%) than ENN B, ENN B_1_, T-2 and HT-2 toxins. Processing can therefore reduce some components of the contamination profile while relatively enriching others in the edible fraction. Moreover, redistribution to non-edible or secondary processing fractions should not necessarily be interpreted as removal from the wider food and feed chain. Hulls, bran and other oat-processing fractions in which mycotoxins may accumulate can be used as animal feed or increasingly valorised as functional ingredients in food applications. Their subsequent incorporation into other products may therefore redistribute the residual mycotoxin burden across food categories and create additional routes of dietary exposure that are not apparent when only the primary edible oat fraction is considered. This is particularly relevant in circular processing systems, where assessing the effect of processing on the overall mycotoxin profile requires consideration of both the main product and the subsequent fate and use of mycotoxin-containing co-products. 

Thermal processing provides an additional example of how processing can reshape the overall mycotoxin profile rather than uniformly reduce all contaminants. Across the studies reviewed, the magnitude of reduction was strongly dependent on both processing severity and mycotoxin identity. Under relatively mild moist-heat conditions, *Fusarium* mycotoxins generally showed high stability; boiling of oat grains produced reductions below 10% for the combined T-2 and HT-2 content, while DON and ZEN were also poorly affected [115,116]. Similarly, roasting of oat flour at 105–120 °C did not significantly alter DON, 15-ADON, ZEN, T-2 toxin, or HT-2 toxin concentrations [79]. This limited effect of thermal treatment was also observed during OBB manufacture, where the sterilisation step did not significantly alter the concentration of DON [125]. In contrast, more severe thermal treatments produced greater but clearly toxin-dependent reductions. Roasting whole oats at 220 °C resulted in complete removal of T-2 toxin, compared with a 43% reduction in HT-2 toxin, while DON and ZEN were reduced by approximately 36–40% and 33–55%, respectively [115,116]. Extrusion produced some of the largest reductions reported, reaching 73–100% for T-2 and HT-2, 73–87% for DON, and 58–100% for ZEN under the conditions evaluated [115,116], although other extrusion studies reported lower reductions, highlighting the strong influence of temperature, moisture, mechanical energy, and residence time. 

Importantly, thermal processing may also alter mixture composition through differences in stability, release, and transformation rather than simply decreasing the concentrations of all compounds. During oat-biscuit production, ZEN was more thermostable than DON-3G, which in turn was more stable than DON; under severe conditions, 15-ADON decreased while DOM-1 increased, and other DON-related transformation products, including isoDON and norDONs, emerged [120]. Moreover, ZEN and its modified forms α-ZEL-14-S and β-ZEL-14-S showed initial increases during baking, consistent with the release of previously inaccessible forms, and final biscuits contained similar DON and DON-3G concentrations but higher ZEN concentrations than initially [120]. These findings demonstrate that an apparent decrease in a parent mycotoxin cannot necessarily be interpreted as an equivalent decrease in the overall mycotoxin burden, because other parent or modified forms may be more stable, become released from the matrix, or be generated or transformed during processing. 

From a risk-assessment perspective, these compound-specific responses are particularly relevant when health-based guidance values are established for groups of parent and modified mycotoxins. For the DON group, a substantial reduction in DON during thermal processing cannot be assumed to produce an equivalent reduction in exposure relative to the group TDI if DON-3G, 3-ADON or 15-ADON show different stability or behaviour. Similarly, preferential degradation of T-2 toxin over HT-2 toxin can change the relative composition of the T-2/HT-2 group without producing a proportional reduction in group exposure, particularly if relevant modified forms persist. Consequently, processing factors derived from individual parent mycotoxins may not accurately represent the change in the toxicologically relevant burden reaching the consumer. Assessment of thermal-processing efficacy should therefore consider, whenever analytical data allow, all forms included within the corresponding group health-based guidance value rather than relying on the reduction in a single marker mycotoxin. 

These broader mixtures require a different interpretation from the DON and T-2/HT-2 groups. Concentrations of toxicologically dissimilar mycotoxins such as DON, ZEN, NIV, ENNs and BEA should not simply be summed or compared with a common TDI, since no common health-based guidance value has been established for such heterogeneous mixtures. Nevertheless, their co-occurrence remains relevant to exposure assessment, and the possibility of combined effects adds further uncertainty when several mycotoxins persist in the same food. At present, however, oat-processing studies provide insufficient toxicological evidence to determine how changes in these heterogeneous mixtures translate quantitatively into changes in combined risk. The most defensible interpretation is therefore to characterise how processing changes the multi-mycotoxin profile while avoiding assumptions of toxicological equivalence where this has not been established.

The strength of the available evidence also varies substantially across processing operations. Evidence for primary processing steps such as cleaning and dehulling is comparatively consistent, with similar patterns reported across several independent studies, whereas evidence for many secondary processing operations remains more limited and heterogeneous. Direct quantitative comparison among studies is further constrained by differences in initial contamination levels, naturally contaminated versus spiked materials, oat matrices and cultivars, processing conditions and severity, analytical coverage, and whether studies report concentration changes or complete mass balances. Consequently, the reduction ranges summarised in this review should be interpreted as process- and study-dependent evidence rather than as universally applicable processing factors. Greater standardisation of experimental design and reporting, together with complete mass-balance approaches, would improve the comparability and strength of future evidence.

Overall, the available evidence indicates that the effect of oat processing on mycotoxin contamination should be interpreted at three complementary levels: changes in individual compounds, changes in the composition of toxicologically defined groups, and changes in the broader multi-mycotoxin profile. Processing may reduce the concentration of several mycotoxins while simultaneously changing their relative contributions to the remaining contamination through differential removal, redistribution, stability, and transformation. Consequently, processing efficacy cannot be fully characterised from reduction factors for individual parent mycotoxins alone, but requires consideration of modified forms, group-based toxicological frameworks where established, and the redistribution of contamination across processing fractions.

## 4. Conclusions

*Fusarium* mycotoxins represent a persistent challenge in oats, whose behaviour along the processing chain is governed primarily by physical redistribution and limited chemical degradation. The evidence reviewed clearly shows that oat processing does not eliminate mycotoxins but rather redistributes them across fractions and products. Early-stage physical operations represent the most effective mitigation steps identified in oat processing. Cleaning and sorting can substantially reduce contamination when effectively removing defective or highly contaminated grains, although their efficacy is variable, whereas dehulling consistently produces some of the largest reductions reported, in some cases exceeding 80–90%. These effects primarily reflect the physical removal and redistribution of mycotoxins into rejected grain fractions, hulls, and other by-products rather than chemical degradation. Importantly, some of these by-products may subsequently enter the animal feed chain, potentially transferring rather than eliminating the mycotoxin burden across the broader food-feed system.

Subsequent operations, particularly milling and fractionation, primarily redistribute mycotoxins among product streams rather than eliminate them, while conventional thermal treatments (e.g., steaming, kilning, baking, or porridge preparation) show limited efficacy due to the high thermal stability of *Fusarium* mycotoxins. In contrast, extrusion emerges as one of the most effective secondary processing operations investigated, achieving substantial reductions under specific conditions, although the mechanisms of degradation remain insufficiently understood. Importantly, mycotoxins can persist throughout processing and be transferred into final oat-based products, including oat-based beverages and cereal-based foods, confirming that processing alone cannot ensure product safety.

Emerging technologies such as cold plasma and rapid detection tools (e.g., NIR-HSI) show promising potential for improving both mitigation and monitoring strategies, although their industrial applicability and impact on mycotoxin transformation require further validation.

Despite these advances, important knowledge gaps remain regarding the behaviour, transformation, and toxicological relevance of mycotoxins along the oat value chain.

## 5. Knowledge Gaps and Future Research Needs

Despite the substantial body of research available, several critical knowledge gaps still hinder a comprehensive understanding of *Fusarium* mycotoxin behaviour along the oat value chain. Although all of these gaps warrant further investigation, three areas appear particularly pressing: (i) elucidating the fate and toxicological relevance of modified and emerging mycotoxins and processing-derived transformation products; (ii) establishing complete mycotoxin mass balances across processing streams, including by-products; and (iii) generating oat-specific evidence for emerging products and insufficiently characterised processing conditions. These priorities are particularly important because they determine whether processing-induced changes in measured mycotoxin concentrations can be reliably interpreted in terms of their implications for exposure and food and feed safety, and whether current evidence adequately reflects the diversity of oat processing practices and products.

The first and arguably most pressing research need concerns the fate of modified and emerging mycotoxins and the identification of transformation products generated during processing. Although modified forms such as DON-3G and T-2-glucoside and emerging mycotoxins such as ENNs and BEA are increasingly reported, their stability and redistribution during processing remain poorly understood, as does the potential conversion of modified forms back into their parent toxins. This is particularly relevant because these compounds may contribute to overall dietary exposure but are not always captured by routine analytical or regulatory frameworks. Furthermore, processing conditions involving heat, pressure, enzymatic activity, water activity, mechanical shear, and interactions with the food matrix may affect the stability and interconversion of modified forms and promote the formation of new degradation and transformation products. The combined effects of these factors are still insufficiently characterised and may explain the considerable variability in mycotoxin reductions reported among studies. Evidence from processes such as extrusion and baking indicates that changes in parent mycotoxin concentrations may be accompanied by the formation, release, or interconversion of other mycotoxin forms and, in some cases, cannot be fully explained by the formation of known degradation products. Consequently, an apparent reduction in the concentration of a parent toxin should not necessarily be interpreted as detoxification, as the fate and toxicological relevance of the fraction no longer detected as the parent compound may remain unresolved. Resolving this uncertainty should therefore be considered a priority, requiring the integration of targeted and non-targeted analytical approaches with structural elucidation and toxicological assessment to determine whether processing genuinely reduces risk or merely changes the chemical forms in which mycotoxins occur.

A second major priority is to move beyond studies based primarily on concentration changes at individual processing steps towards complete mass-balance approaches across the oat processing chain. As demonstrated throughout this review, some of the most effective operations—particularly cleaning, sorting, and dehulling—reduce mycotoxin concentrations in edible oat fractions primarily through physical separation rather than chemical degradation. Consequently, a decrease in one fraction may correspond to the transfer and concentration of mycotoxins in another. This distinction between removal from the edible product, redistribution within processing streams, and actual destruction of the toxin is critical when evaluating the effectiveness of mitigation strategies. Future studies should therefore quantify the recovery of individual mycotoxins and their modified forms across all input and output streams, rather than reporting only percentage reductions in the main edible fraction.

The transfer and accumulation of mycotoxins in oat-processing fractions and co-products represent a particularly important component of this second priority. Hulls, bran, screenings and press cakes may retain or concentrate a substantial proportion of the initial mycotoxin load and may subsequently be used as animal feed or as ingredients for other applications. Therefore, a processing operation that successfully reduces human dietary exposure could simultaneously transfer the mycotoxin burden to another part of the food or feed chain. This issue becomes particularly relevant in the context of circular processing, where oat-derived fractions and co-products may be valorised as functional ingredients and incorporated into multiple food products that are not directly associated with the original oat-processing chain. Such “ingredientization” may extend potential mycotoxin exposure across heterogeneous food categories, adding complexity to aggregate dietary exposure assessment. Consequently, future exposure assessments should consider not only mycotoxin concentrations in conventional oat products but also their occurrence in valorised fractions, their subsequent uses and their inclusion levels in other foods. Similarly, in processes involving solid–liquid separation, such as OBB manufacture, mycotoxins may partition differently between liquid and residual solid fractions. Establishing complete mass balances would provide a more accurate picture of mycotoxin fate and allow the safety of final products, co-products and valorised fractions to be assessed.

The third pressing research need is the generation of oat-specific evidence for emerging products and insufficiently characterised processing conditions. Traditional oat products such as flakes and flour have been comparatively well characterised, whereas considerably less information is available for rapidly expanding matrices such as OBBs, fermented products (e.g., oat yoghurt), oat pasta, plant-based analogues, and other innovative formulations. These products introduce additional operations, including enzymatic hydrolysis, fermentation, fractionation, and solid–liquid separation, which may substantially influence mycotoxin stability, transformation, and transfer. Given the increasing diversity of oat-derived foods, understanding whether these processes remove, redistribute, release, or transform mycotoxins is becoming increasingly important for exposure assessment.

This lack of oat-specific evidence also extends to some more conventional stages of the value chain. Information regarding storage and certain thermal or domestic processing conditions remains limited, and current understanding is sometimes extrapolated from studies conducted in wheat, maize, or other cereals. Although such evidence provides valuable indications, differences in grain structure, composition, and processing behaviour mean that these extrapolations may not always accurately reflect mycotoxin behaviour in oats. Dedicated studies using oat matrices, particularly naturally contaminated material, are therefore needed to establish whether conclusions derived from other cereals can be reliably applied to oats.

Beyond these three priorities, other important knowledge gaps should also be addressed. One of these is the limited consideration of the co-occurrence and combined behaviour of multiple mycotoxins. Most processing studies focus on individual compounds or a limited number of regulated toxins, whereas naturally contaminated oats may simultaneously contain regulated, modified, and emerging mycotoxins. Processing may affect these compounds differently and therefore alter not only their absolute concentrations but also the composition of the mycotoxin mixture reaching the final product. Furthermore, the implications of such co-occurrence for overall exposure and risk assessment remain insufficiently understood. Future studies should therefore increasingly adopt multi-mycotoxin approaches that reflect realistic contamination scenarios and assess how processing modifies both mixture composition and associated risk. 

Addressing these gaps will require integrated and multidisciplinary approaches combining advanced targeted and non-targeted analytical techniques, process engineering, mass-balance studies, and toxicological assessment. Ultimately, future research should provide clearer answers to three fundamental questions: what chemical forms and transformation products result from processing and what is their toxicological relevance; where mycotoxins are transferred or accumulated across processing streams; and to what extent the available evidence adequately represents oat-specific processes and emerging oat-based products. Answering these questions will provide a stronger basis for exposure and risk assessment, support future regulatory developments, and facilitate the design of more effective mitigation strategies across the oat value chain.

## Figures and Tables

**Figure 1:**
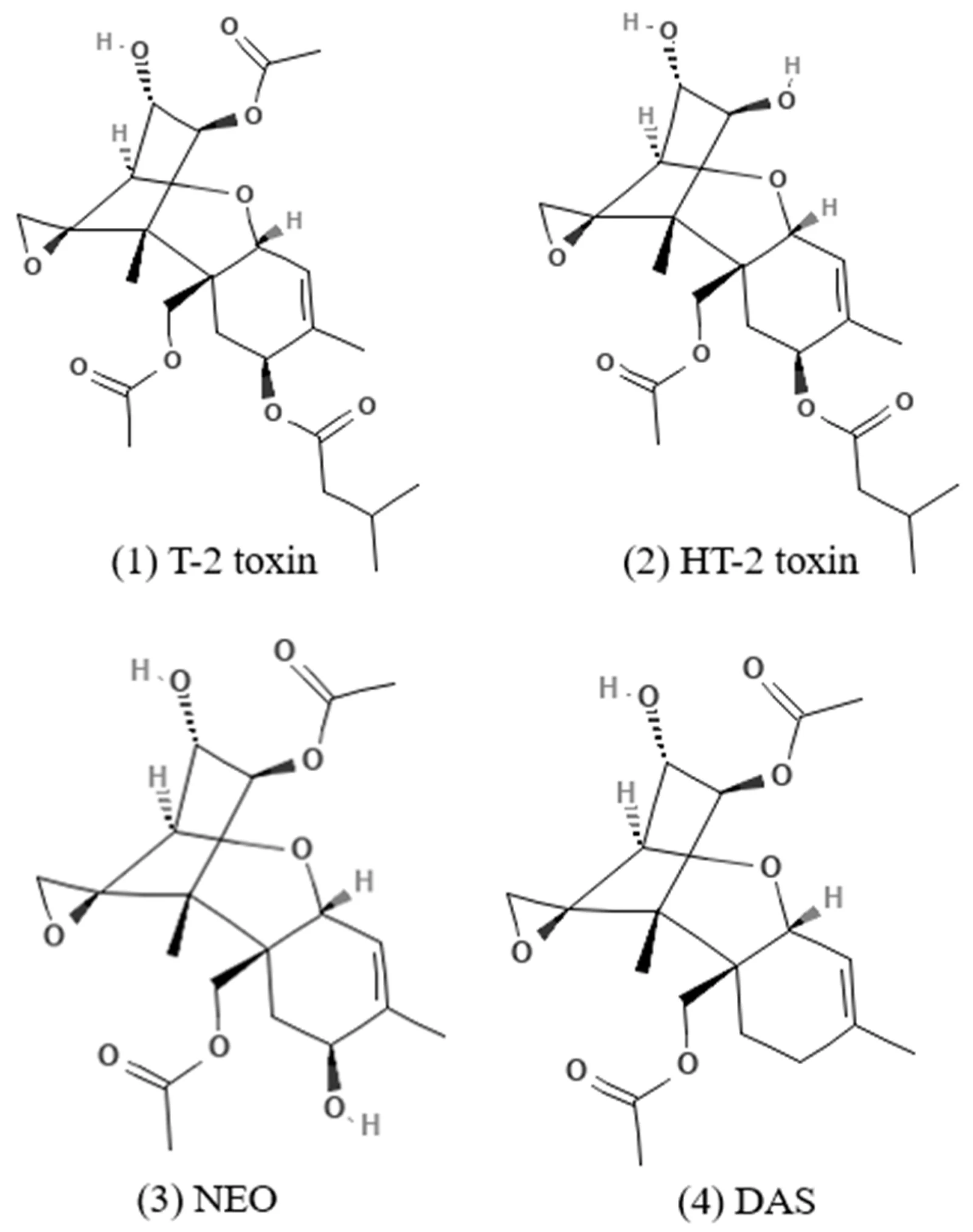
Chemical structures of the type A trichothecenes: T-2 toxin (**1**), HT-2 toxin (**2**), neosolaniol (NEO) (**3**), and diacetoxyscirpenol (DAS) (**4**). Chemical structures were obtained from PubChem (https://pubchem.ncbi.nlm.nih.gov/; accessed on 14 August 2026) and assembled by the authors.

**Figure 2:**
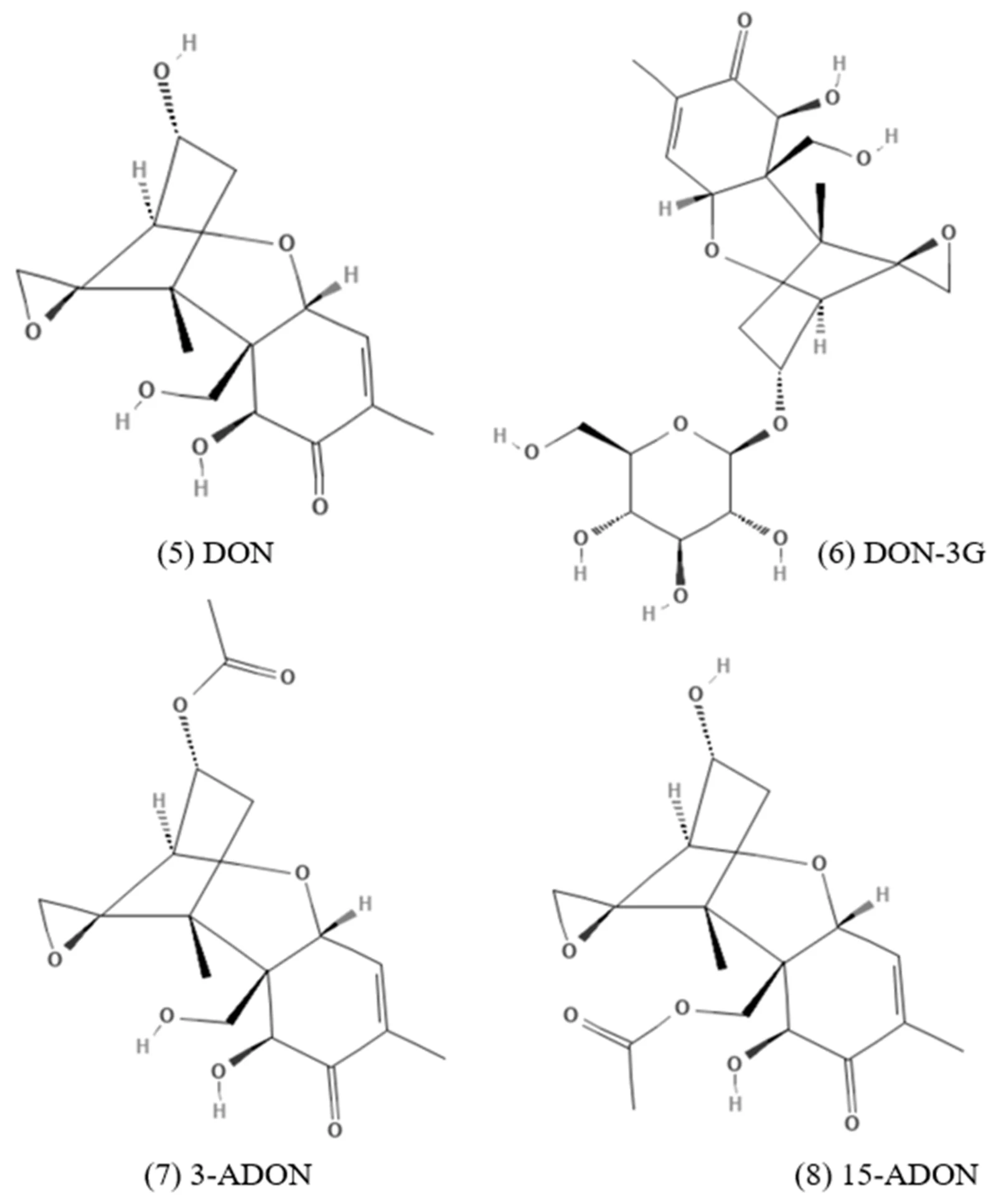
Chemical structures of deoxynivalenol (DON) (**5**) and its modified forms: deoxynivalenol-3-glucoside (DON-3G) (**6**), 3-acetyldeoxynivalenol (3-ADON) (**7**), and 15-acetyldeoxynivalenol (15-ADON) (**8**). Chemical structures were obtained from PubChem (https://pubchem.ncbi.nlm.nih.gov/; accessed on 14 August 2026) and assembled by the authors.

**Figure 3:**
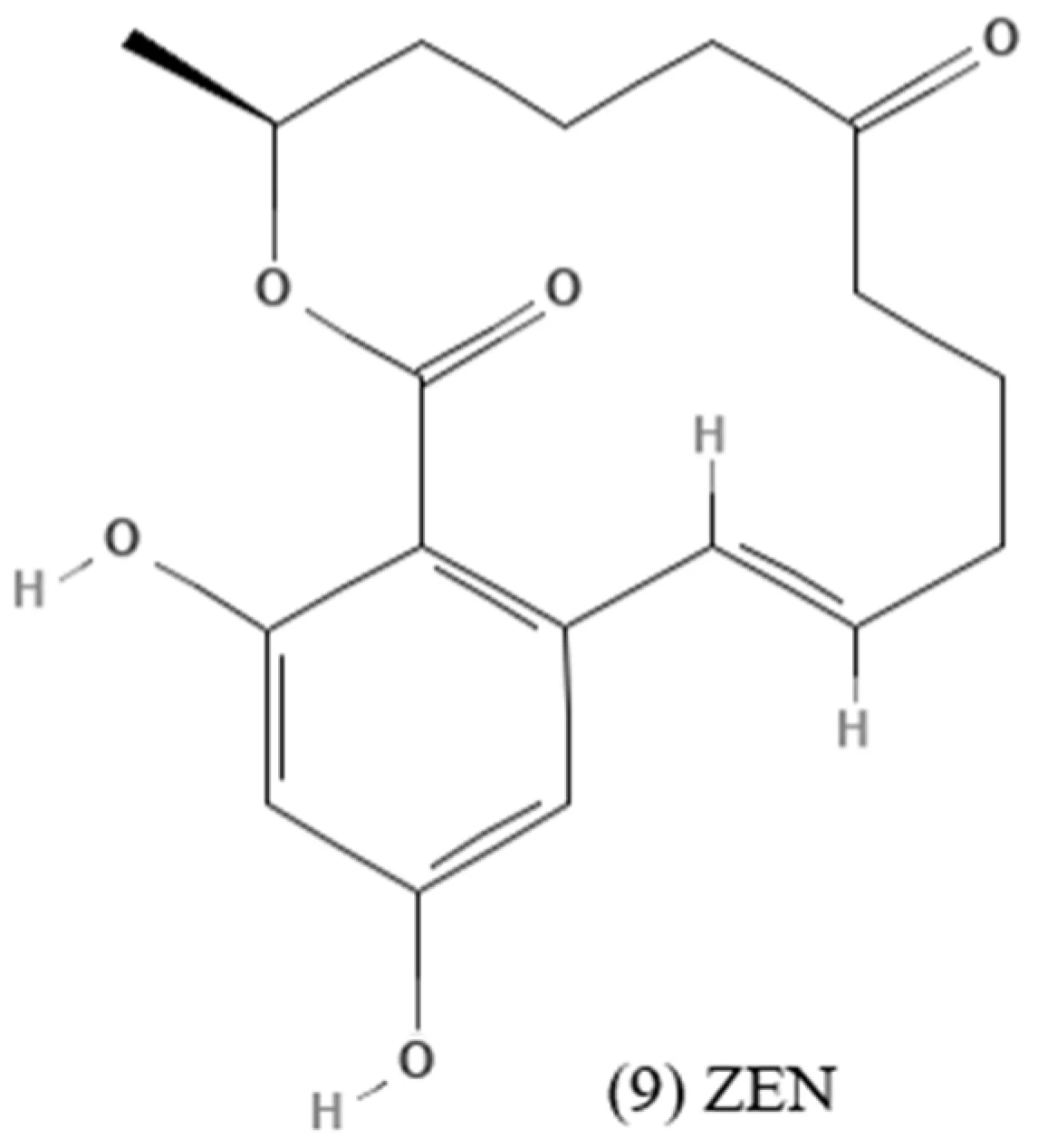
Chemical structure of zearalenone (ZEN) (**9**). Chemical structures were obtained from PubChem (https://pubchem.ncbi.nlm.nih.gov/; accessed on 14 August 2026).

**Figure 4:**
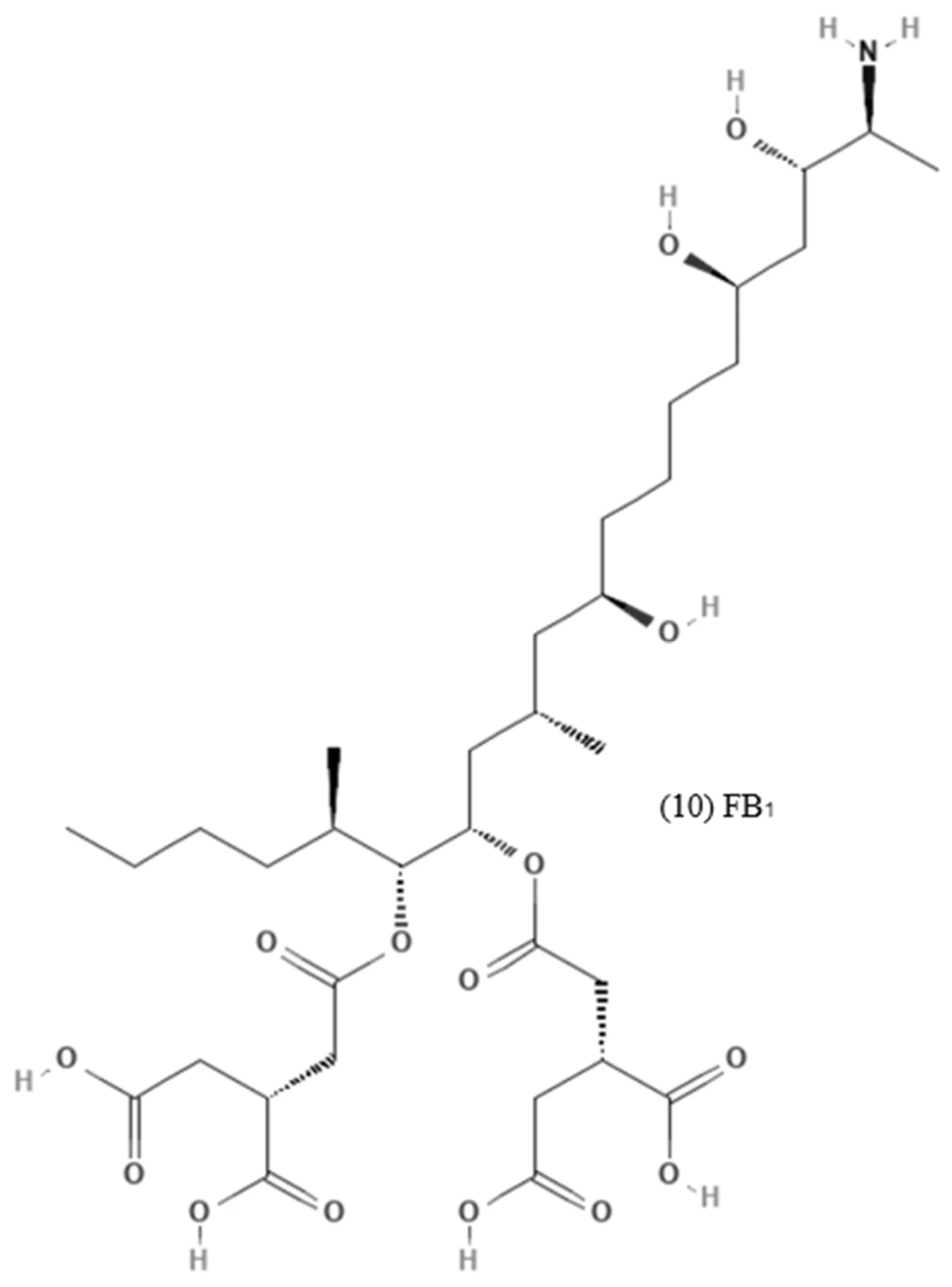
Chemical structure of fumonisin B_1_ (FB_1_) (**10**). Chemical structures were obtained from PubChem (https://pubchem.ncbi.nlm.nih.gov/; accessed on 14 August 2026).

**Figure 5:**
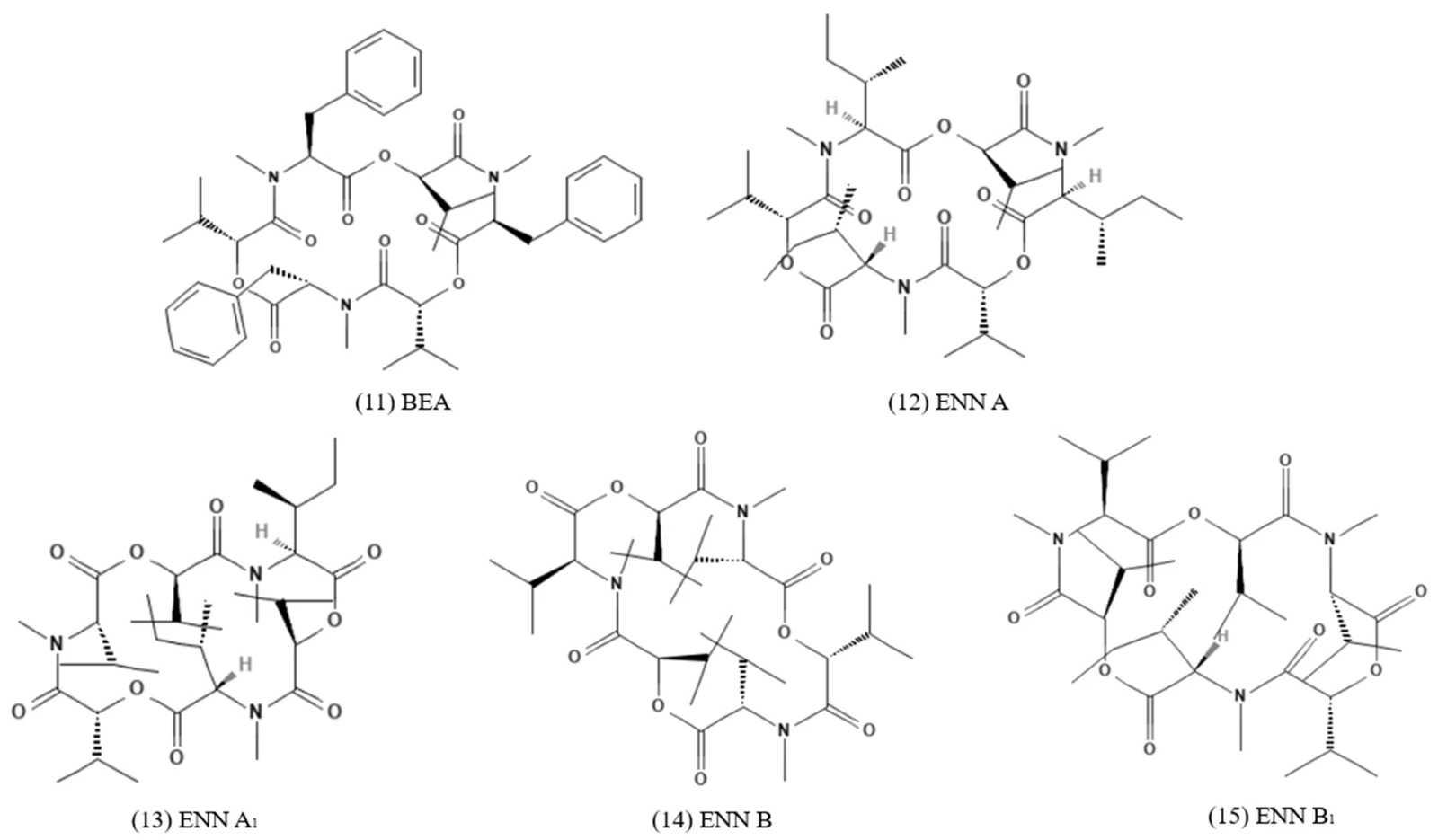
Chemical structures of beauvericin (BEA) and enniatins (ENNs): (**11**) beauvericin (BEA), (**12**) enniatin A (ENN A), (**13**) enniatin A_1_ (ENN A_1_), (**14**) enniatin B (ENN B), and (**15**) enniatin B_1_ (ENN B_1_). Chemical structures were obtained from PubChem (https://pubchem.ncbi.nlm.nih.gov/; accessed on 14 August 2026) and assembled by the authors.

**Figure 6:**
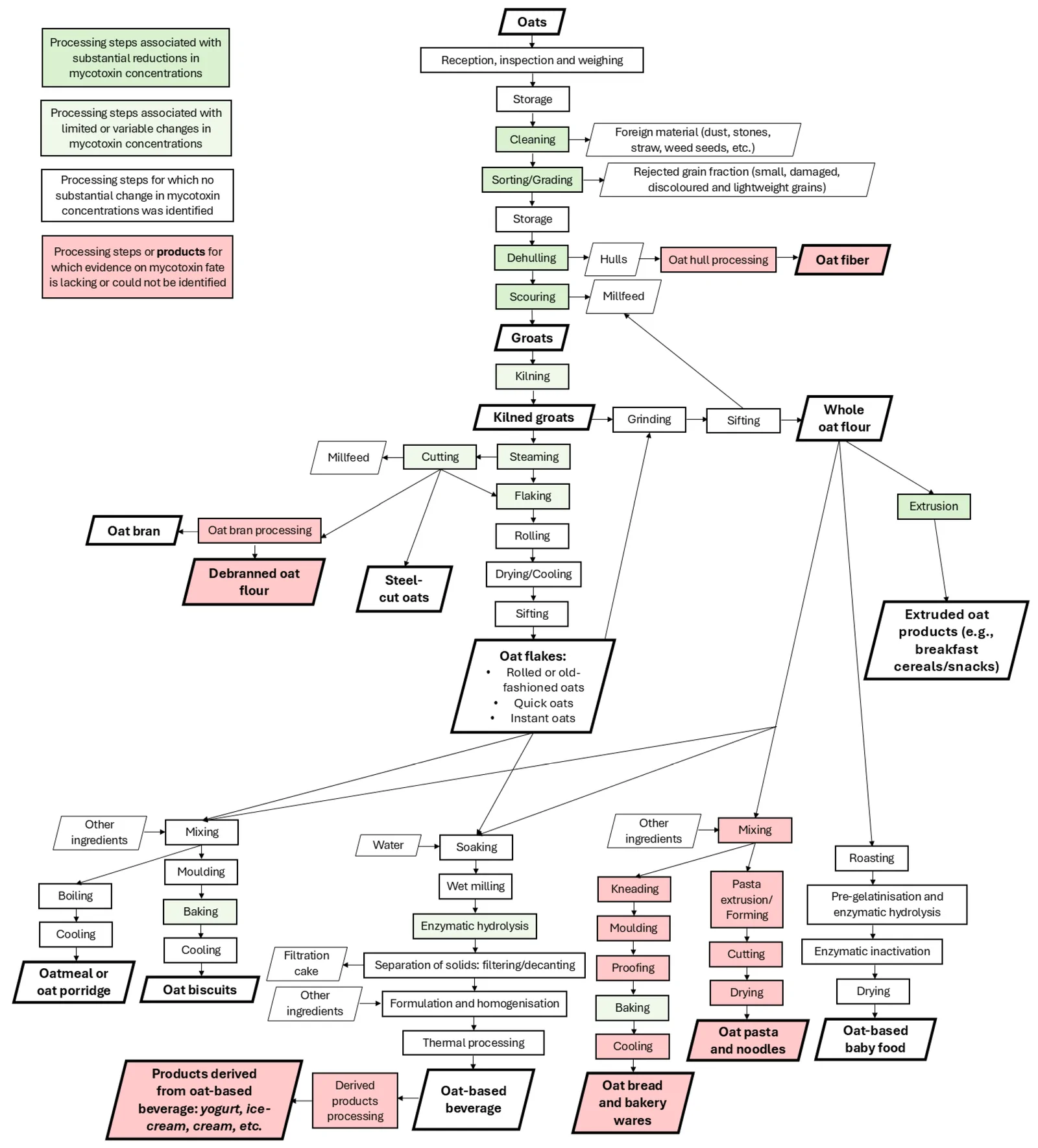
Schematic representation of the oat processing chain—from raw grain to oat-derived food products—highlighting the reported effects of individual processing steps on *Fusarium* mycotoxin concentrations based on the evidence identified in this review. Dark green indicates processing steps associated with substantial reductions in mycotoxin concentrations; light green indicates steps associated with limited or variable changes; white indicates steps for which no substantial change was identified; and pink indicates processing steps or products for which evidence on mycotoxin fate is lacking or could not be identified. Bold text indicates oat products or product fractions obtained throughout the processing chain. Reported effects may vary depending on the mycotoxin, processing conditions, and product matrix.

## Data Availability

No new data were created or analyzed in this study. Data sharing is not applicable to this article.

## Nomenclature

15-ADON	15-acetyldeoxynivalenol
3-ADON	3-acetyldeoxynivalenol
α-ZEL	alpha-zearalenol
α-ZEL-14-S	alpha-zearalenol-14-sulfate
a_w_	water activity
BEA	beauvericin
β-ZEL	beta-zearalenol
β-ZEL-14-S	beta-zearalenol-14-sulfate
CUL	culmorin
DAS	diacetoxyscirpenol
DOM-1	deepoxy-deoxynivalenol
DON	deoxynivalenol
DON-3G	deoxynivalenol-3-glucoside
EFSA	European Food Safety Authority
ENN A	enniatin A
ENN A_1_	enniatin A_1_
ENN B	enniatin B
ENN B_1_	enniatin B_1_
ENNs	enniatins
EU	European Union
FB_1_	fumonisin B_1_
FB_2_	fumonisin B_2_
FHB	Fusarium head blight
FUMs	fumonisins
isoDON	iso-deoxynivalenol
LFD	lateral flow device
LOQ	limit of quantification
NEO	neosolaniol
NIR-HSI	near-infrared hyperspectral imaging
NIV	nivalenol
norDON	nor-deoxynivalenol
OBB	oat-based beverage
TDI	tolerable daily intake
UK	United Kingdom
USA	United States of America
ZEN	zearalenone
ZEN-14-S	zearalenone-14-sulfate
